# Toward Practical Single‐Molecule/Atom Switches

**DOI:** 10.1002/advs.202400877

**Published:** 2024-05-29

**Authors:** Xiaona Xu, Chunyan Gao, Ramya Emusani, Chuancheng Jia, Dong Xiang

**Affiliations:** ^1^ Institute of Modern Optics and Center of Single Molecule Sciences Nankai University Tianjin Key Laboratory of Micro‐scale Optical Information Science and Technology Tianjin 300350 China

**Keywords:** atomic switches, molecular devices, molecular electronics, molecular switches

## Abstract

Electronic switches have been considered to be one of the most important components of contemporary electronic circuits for processing and storing digital information. Fabricating functional devices with building blocks of atomic/molecular switches can greatly promote the minimization of the devices and meet the requirement of high integration. This review highlights key developments in the fabrication and application of molecular switching devices. This overview offers valuable insights into the switching mechanisms under various stimuli, emphasizing structural and energy state changes in the core molecules. Beyond the molecular switches, typical individual metal atomic switches are further introduced. A critical discussion of the main challenges for realizing and developing practical molecular/atomic switches is provided. These analyses and summaries will contribute to a comprehensive understanding of the switch mechanisms, providing guidance for the rational design of functional nanoswitch devices toward practical applications.

## Graphical abstract

This review highlights the key developments in the fabrication and application of molecular/atomic switching devices, aiming to provide a comprehensive understanding of the working principles and elucidate the factors that influence their performance. A critical discussion of the main challenges for realizing and developing practical molecular/atomic switches is provided as well.

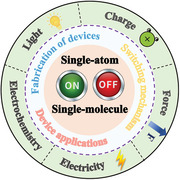

## 1 Introduction

Molecular electronic devices employ individual organic molecules or small organic molecular aggregates to create, measure, and analyze electronic and optoelectrical processes at the molecular level. This approach enables the development of novel molecular or atomic‐based electronic and optoelectronic devices, including molecular switches,^[^ ^1^, ^2^, ^3^, ^4^ ^]^ molecular diodes,^[^ ^5^, ^6^, ^7^ ^]^ and molecular transistors.^[^ ^8^, ^9^, ^10^ ^]^ Molecular switches, as fundamental components in modern logic and memory circuit design, have garnered significant interest. These switch's processes can store digital information by altering molecular conductance and directing signals within the electronic logic circuits. Typically, molecular‐scale switches achieve switching functionality through reversible transitions between at least two stable states, each possessing unique physical and chemical properties due to specific molecular geometries or electronic distributions. In contrast, atomic switches are relatively straightforward to operate because they only need to connect or disconnect metal atoms or rearrange them.^[^ ^11^ ^]^ Advancements in micro/nanofabrication techniques and the discovery of materials with unique geometrical and chemical/physical properties have spurred exploration into various molecular systems, device fabrication methods, and switching devices, such as photoelectric switches and charge (or force) controlled switches. Concurrently, a range of atomic switches has also emerged in scientific research.

In this review, we discuss the fundamental elements and working principles of single‐molecule/atom switch devices, which have gained significant attention in molecular electronics. The performance of the devices is influenced by multiple factors, such as electrode materials, type of molecules, and fabrication techniques. Metal electrodes are widely employed in these devices due to their high conductivity and ease of integration with molecules.^[^ ^12^, ^13^, ^14^ ^]^ In contrast, the carbon‐based electrode shows unique advantages in stability and compatibility with biological molecules.^[^ ^15^ ^]^ Regarding the molecules, key factors include molecular conformation, frontier orbital energy, charge transport pathway, interfacial coupling strength, and electronic spin state. The functionality of these devices is based on molecular conductance switching, which is achieved through exposure to external stimuli, including light,^[^ ^16^, ^17^ ^]^ electric field,^[^ ^18^, ^19^ ^]^ and force.^[^ ^20^, ^21^, ^22^, ^23^ ^]^ This necessitates using molecules with at least two stable isomers, capable of transitioning between these states.^[^ ^24^ ^]^ Designing and synthesizing suitable molecules is crucial for developing efficient switching devices. Generally, these molecules can be categorized into two types: those with multiple stable‐state isomers that exhibit altered physical properties upon stimulation, and those that undergo conformational changes, such as transitioning between open‐loop and closed‐loop states under external influence.^[^ ^2^, ^24^, ^25^, ^26^ ^]^ Overall, this review aims to provide a comprehensive understanding the operating principles, elucidating the factors that influence their performance and the potential wide applications of these innovative technologies.^[^ ^27^, ^28^, ^29^ ^]^

Molecular and atomic switching devices have gained more attention from researchers in the last few decades due to the tremendous breakthroughs in the field of micronanoscale molecular electronics.^[^ ^16^, ^21^, ^24^ ^]^ In recent years, there have also been many excellent review articles related to this topic while focused on the molecule machine and motors.^[^ ^30^, ^31^, ^32^, ^33^ ^]^ This review aims to provide a comprehensive summary of the significant achievements in the field of molecular and atomic switches, with an emphasis on complex molecular switch device systems. Three primary viewpoints will be covered in our discussion of these systems: device materials, device preparation and fabrication, and switching mechanisms. Our goal is to help readers gain a deeper understanding of the latest developments in this field while also highlighting the challenges and opportunities that lie ahead. Nano microscale conductance molecular switches are an emerging area of research with many challenges but they also hold immense potential for future applications in science and technology. Consequently, we will also provide a brief outlook on the future development of molecular and atomic switches, emphasizing their potential practical application.

## 2 Fabrication of Molecular Devices

Molecular and atomic switching devices are integral to the functionality of numerous electronic systems, with their performance being primarily influenced by three key factors: electrode materials, anchoring groups, and fabrication methods. The choice of electrode material is a crucial part as it directly influences the efficiency of a switching device. For this reason, it is essential to understand the characteristics of various electrode materials to optimize device performance. An additional critical aspect of the performance of molecular devices is the coupling between electrodes and molecules, which is governed by anchoring groups. Furthermore, the fabrication method that is used to create molecular devices is also very important since it determines key performance factors like stability and repeatability. In the following sections, we will provide a comprehensive examination of these three pivotal aspects in the fabrication of molecular and atomic switching devices. By delving into the complexities of electrode materials, anchoring groups, and fabrication methods, this review intends to aid in the development of more efficient molecular devices for a wide range of applications.

### 2.1 Electrodes Materials

Electrode materials play a pivotal role in determining the performance, stability, and scalability of molecular devices, as they create interfaces with molecular components and facilitate charge transport. This chapter summarizes the challenges associated with choosing suitable electrode materials for different types of molecular devices, along with potential solutions and strategies to overcome these obstacles. Metal electrodes such as Au, Ag, and Pt have been the most commonly employed over the past few decades due to their unique properties.^[^ ^12^, ^13^, ^14^, ^34^, ^35^, ^36^, ^37^, ^38^ ^]^ Especially, Pt shows advantage in stability (thus the breakdown field of the molecule junction could be improved) since it can withstand a larger wind force.^[^ ^39^, ^40^ ^]^ To create a strong and stable molecular junction, covalent bonds such as Au‐S, are usually used to connect metal electrodes with molecules. However, there are limitations associated with metal electrodes, such as challenges in manufacturing nanoscale gaps, uncertainties in the number of molecules between electrodes due to uncontrollable geometrical configurations at the contact points, and ambiguities in linkage chemistry. These issues have prompted researchers to investigate alternative electrode materials to facilitate controllable fabrication and interfacial regulation of molecular junctions, ultimately aiming to develop functional molecular‐scale devices.

Carbon‐based electrodes, such as carbon nanotubes (CNTs) and graphene, have emerged as promising candidates for molecular devices due to their unique geometric features, chemical composition, and natural affinity with organic molecules.^[^ ^41^, ^42^, ^43^, ^44^ ^]^ In comparison to metallic electrodes, these materials perform better at stabilizing interfacial linkages.^[^ ^45^, ^46^ ^]^ Particularly, graphene electrodes offer a number of distinct advantages. First, they are naturally compatible with organic and biological molecules. Second, graphene electrodes exhibit remarkable stability and chemical flexibility due to the π‐conjugated skeletons. Moreover, these electrodes can be fabricated on a wide range of substrates using bottom‐up chemical approaches with high precision.^[^ ^24^ ^]^ By combining molecular engineering principles, the electrode‐molecule bonding can be readily tuned without compromising robustness and stability, enabling the adjustment of molecule‐electrode coupling strength. This capability is crucial for obtaining intrinsic molecular properties and achieving desired functions.^[^ ^44^, ^47^, ^48^ ^]^ Specifically, the atomic thickness of graphene is well‐matched to the dimensions of small molecules, and the building blocks of sp^2^‐hybridized carbon atoms exhibit rich chemistry and well‐defined interfaces when interacting with these molecules. Consequently, graphene is anticipated to become a competitive alternative electrode material for the fabrication of molecular devices.

Introducing new electrode materials into the realm of molecular electronics holds great significance, as it offers a plethora of opportunities to construct interfacial connections, fine‐tune energy level alignments, and establish innovative device architectures. These advancements are essential for driving the integration of nanostructured devices and enabling single‐molecule circuits with promisingly higher yields and lower power dissipation. Conductive polymers, for instance, have been employed as electrodes in molecular junctions, which can potentially decrease the electrode width to the molecular scale and facilitate the fabrication of feasible single‐molecule integrated circuits.^[^ ^49^ ^]^ Moreover, a variety of novel 2D materials have emerged following the discovery of the groundbreaking material graphene. These materials, including graphene^[^ ^50^, ^51^ ^]^ and graphdiyne,^[^ ^52^ ^]^ exhibit unique properties, rendering them highly promising for applications in next‐generation nanoelectronics.^[^ ^53^ ^]^ The development of nanoscale electrodes from these novel materials provides numerous possibilities for constructing a robust molecular electrode interface. This advancement can propel the integration of molecular electronic circuits and the evolution of molecular electronic devices by adjusting energy level arrangements or forming new bonding connections.

### 2.2 Anchor Groups

The interfacial coupling has been demonstrated to be crucial in the development of nearly all types of molecular devices, as it directly impacts the conductivity of nanocircuits.^[^ ^54^, ^55^, ^56^ ^]^ Therefore, optimizing the anchoring groups employed in constructing molecule‐electrode linkages is crucial for determining the interfacial coupling strength and aligning the energy levels of molecular orbitals with the Fermi levels of electrodes.^[^ ^57^ ^]^ It is generally believed that increasing the electron delocalization of terminal groups can enhance the interfacial coupling strength, thus reducing the contact resistance for intrinsic charge transport within molecular junctions.^[^ ^25^ ^]^ However, selecting anchoring groups for the creation of molecular switches should prioritize enhancing switching capabilities over merely increasing conductivity. The appropriate choice of interface groups can contribute to improved stability, controlled interface coupling, and reduced interference from interface polymorphism on switching performance. The anchoring groups in Au–molecule–Au junctions have been extensively investigated, enabling the adjustment of electronic coupling between molecules and electrodes, ultimately controlling the interfacial properties of molecular junctions. This research has led to a deeper understanding of several anchoring systems, such as thiol (‐SH) and amine (─NH_2_) anchors. Additionally, new systems with multiple surface attachment sites have been developed, including isocyanide (─N≡C),^[^ ^58^ ^]^ carboxylic acid (─COOH),^[^ ^59^ ^]^ pyridine,^[^ ^60^ ^]^ Nitrile (─C≡N),^[^ ^56^ ^]^ thioether (─SMe),^[^ ^61^ ^]^ thiocyanate (─NCS),^[^ ^62^ ^]^ dimethyl phosphine (─PMe_2_),^[^ ^61^ ^]^ diphenylphosphine (─PPh_2_),^[^ ^63^ ^]^ thiocarboxylic acid (─CS_2_H).^[^ ^64^ ^]^ These systems are anticipated to be less susceptible to the variations in local microstructure at the interface, thereby resulting in more robust and reproducible devices.

Additionally, the Dithiocarbamate (─NCS_2_H) groups can form stable molecular junctions with Au electrodes,^[^ ^65^ ^]^ exhibiting contact resistance two orders of magnitude lower than thiolates. Trimethyl tin (─SnMe3) groups enable in situ covalent Au‐C σ bonding by directly connecting the molecule's end with the electrode's gold atoms.^[^ ^66^ ^]^ Fullerene (C_60_) can also serve as an anchoring group, with strong coupling between C_60_ moieties and Au electrodes achieved through a hexagonal ring or double bond, due to partial charge transfer and strong hybridization effects.^[^ ^25^, ^67^ ^]^ Benzene rings with strained structures can form stable Au‐C bonds as well in the presence of electron‐donating substituents.^[^ ^25^ ^]^

### 2.3 Fabrication Techniques

One of the most critical processes in establishing molecular electronic testing platforms is fabricating nanogaps for assembling molecules. Various fabrication methods have been developed to construct electrode‐molecule‐electrode junctions, depending on the properties of different electrode materials, molecules, and environments.^[^ ^68^ ^]^ Fabricating a nanoscale electrode gap is the primary objective of these methods, which enables a single or few numbers of molecules to effectively bridge the electrodes, establishing a reliable molecular junction and forming a closed circuit. Several fabrication techniques have been employed to produce nanoscale metal electrodes, including scanning probe microscopy (SPM),^[^ ^69^, ^70^ ^]^ mechanically controllable break junctions (MCBJ),^[^ ^71^ ^]^ electromigration breakdown junctions,^[^ ^72^ ^]^ electrochemical deposition junctions,^[^ ^73^ ^]^ the lift and float approach,^[^ ^74^ ^]^ and on‐wire lithography.^[^ ^75^ ^]^ Each method offers unique characteristics for fabricating molecular junctions. We will provide a detailed overview of selected methods for ref. [24].

**Figure 1:**
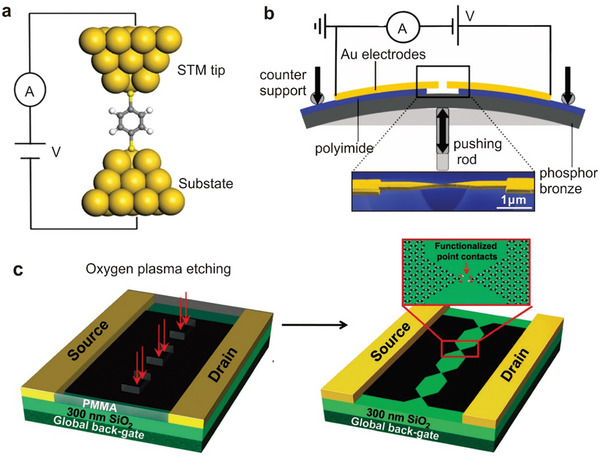
Methods for single‐molecule junction fabrication. a) Mechanism of the STM setup. Reproduced with permission from ref. [92] Copyright 2016, Springer Nature. b) Mechanism of the MCBJ setup. Bottom: Scanning electron micrograph of an MCBJ device. The scale bar shows that the suspended bridge is about 1 µm in length. Reproduced with permission from ref. [93] Copyright 2011, Beilstein‐Institut. c) Fabrication of indented graphene point contact arrays by DLL. Left: Oxygen plasma through a serrated (PMMA) window which is determined by electron beam lithography for cutting grapheme sheets accurately. Right: The point contacts of serrated graphene were generated by oxidative cutting and functionalized by carboxylic acid end groups. They were also separated by intervals of several nanometers. Reproduced with permission from ref. [90] Copyright 2012, Wiley‐VCH GmbH.

The SPM break junction, developed in the 1980s, was the earliest technique for investigating single molecules. SPM includes scanning tunneling microscopy (STM),^[^ ^70^, ^76^ ^]^ and atomic force microscope (AFM),^[^ ^69^, ^77^ ^]^ both widely used in the fabrication and characterization of molecular electronic devices. A straightforward and effective method for constructing molecular junction fractures is scanning tunneling microscope‐controlled break junctions (STM‐BJ).^[^ ^78^, ^79^ ^]^ The formation of STM‐BJ molecular junctions involves two steps. First, the STM‐BJ electrodes approach until a closed circuit of several gold atoms is formed, and then contact is gradually separated. When the electrodes separate a certain distance, the target molecules may attach to the electrodes via chemical bonds or physical adsorption, to form a molecular device, as shown in **Figure** **1**a. This method enables tens of thousands of electrical statistical tests on the same molecular solution. The advantages of STM‐BJ include simplicity, high junction efficiency, fast test speed, and diverse research systems due to the unique electrode construction method. The dynamic bonding process facilitates single molecular junction formation and studies of individual molecular physical properties. Replacing electrodes with different materials allows for heterojunction studies. However, the dynamic process may lead to device structure randomness and uncontrollable geometric contact morphology between the molecular wire and electrode accompanied by mechanical vibrations. Typically tested at room temperature, STM‐BJ may be affected by the surrounding environment and electrode‐compression‐time.^[^ ^80^ ^]^ In contrast, AFM detects the minute force between the tip and the molecule through cantilever deformation and optical path amplification. Conductive probe‐AFM (CP‐AFM) technology can be achieved by coating a conductive layer on the silicon or silicon nitride tip of the AFM, allowing control of the contact force between molecules and electrodes while conducting electrical tests.

Another commonly used technique is the MCBJ technique, which was introduced by Moreland and Ekin in 1985 to study the superconductor tunneling properties.^[^ ^81^, ^82^ ^]^ Later, Muller and van Ruitenbeek utilized MCBJ technology to investigate metal electrode atomic point contact.^[^ ^71^ ^]^ In 1997, Reed et al., as the first one, measured single molecule characteristics using the MCBJ technique.^[^ ^83^ ^]^ Since then, MCBJ has been widely applied to characterize the dynamic performance of single molecular junctions due to its unique advantages in stability.

As illustrated in Figure 1b, a typical MCBJ setup consists of three parts: a suspended metal bridge with a constriction, micro‐fabricated on the substrate; a push rod to break the nanostructure; and a counter support to bend the substrate. Initially, a small piece of metallic wire with a notch in the middle is fixed onto a flexible substrate. The substrate can be bent using a three‐point countersupport configuration. A vertical movement of the push rod, which can be accurately controlled by an operator, exerts force on the flexible substrate. The metal wire elongates as the substrate bends, resulting in the cross‐section at the notch to shrink. When the metal wire is stretched further, it finally breaks the metal wire completely at the notch point, automatically generating two clean‐facing electrodes. The distance between the electrodes can be accurately controlled by bending or relaxing the substrate due to the amazing attenuation factor.^[^ ^84^, ^85^ ^]^ Based on the typical MCBJ which uses pure metal as an electrode, Zhao et al., further develop an optical fiber‐based break junction (F‐BJ) technique, in which the optical fiber covered with a metal layer can work as both electrodes and optical waveguide, making it feasible to address the electrical and optical signals of single‐molecule junctions simultaneously.^[^ ^86^ ^]^Although the MCBJ technique is very useful for fundamental investigation in the different research fields and can be easily combined with other techniques, it is not facile to fabricate highly integrated molecule‐based devices because of the constraint of the push rod components and the three‐point apparatus with bending substrate.

Electron beam lithography (EBL) is a technique used to create high‐resolution patterns in a thin layer of resist material, typically composed of polymers or other organic compounds.^[^ ^87^, ^88^ ^]^ The technique uses a focused beam of electrons to selectively expose areas of the resist, which can then be selectively removed using a solvent in a process known as development. This technique allows for the creation of intricate structures in the resist that can subsequently be transferred to the substrate through etching. During the EBL process, the focused beam of electrons scans across the surface of the resist material point by point, selectively exposing the whole target area. The energy of the electrons is carefully controlled to ensure that they penetrate the resist material to a specific depth, creating a well‐defined pattern that can be transferred to the substrate. The electron beam modifies the resist's solubility, allowing for the selective removal of either the exposed or unexposed areas of the resist material during the development process. This produces a patterned resist layer that can be utilized as a mask for subsequent etching of the substrate. Overall, EBL is a powerful technique for the creation of high‐resolution patterns in a wide range of materials, including semiconductors, metals, and polymers. Its versatility and high resolution make it a valuable tool for a wide range of applications in nanotechnology, including the fabrication of micro‐ and nano‐electronic devices, sensors, and optoelectronic components.

Due to the unique performance requirements of molecular devices, several electron beam etching derivatives have emerged, including dotted line lithography (DLL), plasma etching, and so on. DLL is a highly effective method for manufacturing nano‐gaps at molecular scales, with the ability to produce carboxylic acid‐terminated graphene point contact arrays.^[^ ^89^ ^]^ As shown in Figure 1c, the procedure starts with designing a CAD file featuring a 5 nm wide dotted line, which is then exposed to the electron beam and spaced at 40 nm intervals. This exposure creates an indented window in a spin‐cast layer of poly (methyl methacrylate) (abbreviation: PMMA) using ultra‐high‐resolution EBL. Subsequently, a high‐quality single‐layer graphene sheet is locally cut through the open window via oxygen plasma ion etching. Exploiting the gradual etching and undercutting of the PMMA allows for the formation of narrow gaps between indented graphene point contact arrays. These contacts interact with conductive molecules derivatized with amines, forming stable molecular devices through amide linkages. The DLL technique significantly simplifies the device fabrication process and results in a high yield of stable molecular devices. The combination of ease in fabrication and device stability, graphene‐molecule single‐molecule junctions are a promising platform for the investigation of molecular electronics. Guo's group utilized the DLL process to fabricate graphene point contact arrays by inventing etching techniques within the masked region of the graphene. Subsequently, robust single‐molecule electronic devices were fabricated based on the indented graphene point electrodes, and a series of related studies were conducted.^[^ ^45^, ^90^, ^91^ ^]^ This work demonstrates the potential for integrating multiple functionalities into single molecular devices.

Nanoimprint lithography (NIL), mainly including imprint lithography and stamp‐printing, is a widely used method to produce multi‐molecular junctions simultaneously.^[^ ^94^ ^]^ Both methods effectively reduce mechanical vibration and enhance thermal stability, allowing for long‐term *I*‐*V* characteristics monitoring to analyze electrical properties. Imprint lithography normally utilizes electron beam lithography and reactive ion etching to create an imprinting mold. Typically, this mold consists of thermally grown silicon oxide patterns on a silicon substrate, formed using an electron beam writer and oxygen reactive ion etching (RIE). The SiO_2_ surface is then patterned and etched to produce micron‐level lines with a pad at each end. Each mold contains hundreds of nanowires that are heated and pressed onto a coated substrate, transferring the pattern from the mold to the PMMA layer. The mold and device substrate are then cooled to room temperature before separation. Subsequently, oxygen RIE is used to remove residual PMMA and expose the SiO_2_ surface. Finally, a metal layer is evaporated onto the substrate, and an acetone stripping process removes un‐patterned regions, leaving the metal nanowire and its micro‐level connection with the contact pad.^[^ ^95^ ^]^

Mirkin's group introduced the on‐wire lithography method,^[^ ^96^ ^]^ which utilizes the difference in corrosion resistance of materials and combines porous template electrochemical deposition with wet etching technology. This method is based on molecular junction techniques initially employed in on‐wire lithography.^[^ ^75^ ^]^ They later simplified the molecule‐metal interface by fabricating a self‐assembled monolayer (SAM) in‐wire molecular junction through the direct reduction of adsorbed metal anions on the SAM surface. However, they encountered challenges due to the complex process and uncertainty of the metal‐molecule interface. To address the issues of metal penetration and molecular layer thermal ablation caused by electrode deposition, a metal nanowire was used as the top electrode and assembling electrode arrays in a cross structure was established.^[^ ^97^ ^]^

Although remarkable strategies for the fabrication of nanogaps are proposed, wafer‐compatible nanogaps with freely adjustable gap sizes are rarely reported. Herein, Zhao et al. proposed an approach for constructing in situ adjustable metal gaps that allow angstrom modulation resolution by employing either a lateral expandable piezoelectric sheet.^[^ ^98^ ^]^ Briefly, they take a longitudinal polarized rectangular piezoelectric ceramic sheet as the key substrate, and the upper and bottom surfaces of the substrate are coated with a silver film. The piezoelectric ceramic substrate sandwiched between the two silver electrodes will have a lateral deformation when a drive voltage is applied to the silver electrodes, which can make the nanostructure fixed on the substrate break resulting in a horizontally adjustable nanogap. The wafer‐compatible nanogaps and in‐plane dynamical break‐junctions provide a potential approach to fabricating highly compacted devices employing single molecules as a building block. Furthermore, Zhang et al., within the same group proposed a light‐controlled in‐plane nanogap approach.^[^ ^99^ ^]^ They demonstrated that in‐situ adjustable nanogaps arrays with sub‐angstrom precision can be fabricated via laser irradiation on the substrate, which supports the electrode pairs. They demonstrated that the atomic atomic‐contact switches can be realized, and the direction of the switching can be reversed by the control of laser irradiation position. They further revealed that the light‐controlled switches originate from the thermal expansion of the substrate upon light irradiation. Furthermore, the nanogap's size can be continuously changed by varying the incident laser power gradually. In this way, they provide a novel break junction technique to address the properties of single‐molecule junctions.

The aforementioned fabrication methods for single molecular devices provide a foundation for constructing various molecular switching devices. However, they also have inherent limitations more or less, such as mechanical stability, thermal stability, construction process cost (time and money), precision, and stability and controllability of the electrode gap. Thus, there is an urgent need to develop novel technologies for manufacturing nanometer gaps in a controllable manner and to conduct comprehensive research on devices from multiple perspectives. Notably, it will be a great challenge to fabricate fully controllable atomic‐scale nanogap due to the movement of gold atom above the electrode surface.^[^ ^100^, ^101^ ^]^Anyway, scalable technique is essential for advancing the rigorous scientific investigation of molecular‐level electronic technology and fostering potential large‐scale industrial applications.

## 3 Photoisomeric Molecular Switches

The photoisomer switch is a fascinating and versatile molecular system that has captured the attention of researchers in a wide range of scientific disciplines, from chemistry and materials science to biology and medicine. At its core, the photoisomer switches are created by manipulating light‐responsive molecules to transit between high and low conductance states via light stimulation. Photoisomerization molecular devices can be broadly categorized based on two main aspects: the types of photo isomeric molecules and the types of optical switches. The types of photoisomeric molecules can be classified based on their molecular structures and photochemical properties. On the other hand, the types of optical switches used in photoisomerization molecular devices are based on distinct physical mechanisms.

### 3.1 Photoisomeric Molecules

Various molecular functional switch devices have been reported, offering a diverse range of molecules to switch.^[^ ^102^ ^]^ Photochromic molecules have gained significant interest for molecular switches, as their properties can be controlled through photochromic reactions. The backbone of photochromic molecules in molecular junctions for photo‐switches, which leverage the reversible photoisomerization reaction of molecules through excited states and transitions between two states. During the transition process, the isomer exhibits a range of unique characteristics, such as absorption and fluorescence properties, refractive index, crystal structure, hydrophobicity, magnetic properties, and electrical conductivity.^[^ ^103^, ^104^, ^105^ ^]^ Sensitive experimental techniques, including fluorescence and surface‐enhanced Raman spectroscopy (SERS), provide insights into photochromic transitions at the single‐molecule level.^[^ ^106^, ^107^ ^]^ Motivated by the potential applications of molecular switches, various molecules with switching capabilities have been proposed for use in molecular devices. Here are several typical molecular backbones based on photochromic properties, as outlined in **Table** **1**.

**Table 1:** Backbone of molecules for photoswitches.

Molecules	On State	Off State	Reference
Diarylethenes	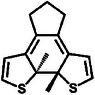	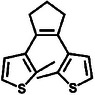	[3, 108, 109, 110]
Azobenzene	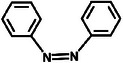	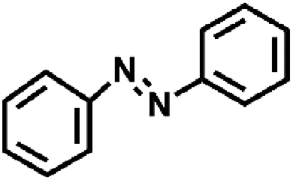	[111, 112, 113, 114, 115]
Spiropyrans	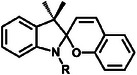	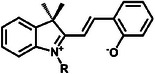	[116, 117, 118, 119]
Dihydropyrene	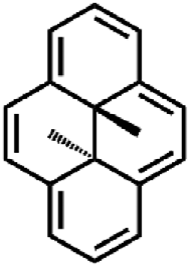	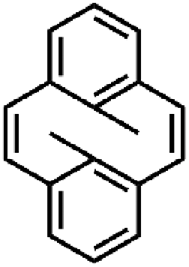	[120, 121, 122, 123, 124]

### 3.2 Diarylethene Switches

Diarylethene has been extensively reported as photoswitching units for single‐molecule optical switching devices.^[^ ^1^, ^108^, ^109^, ^110^, ^125^, ^126^, ^127^, ^128^, ^129^, ^130^, ^131^, ^132^ ^]^ These compounds exhibit exceptional thermal stability, fatigue resistance, high cyclization, and light chemical sensitivity. Diarylethene can undergo transitions between open‐ring and closed‐ring states upon exposure to different wavelengths of absorption spectroscopy light,^[^ ^130^ ^]^ demonstrating effective photochromic behavior in both solutions^[^ ^16^ ^]^ and solid states.^[^ ^110^, ^133^, ^134^ ^]^ Similar to diarylethylene, chiral nematic liquid crystals (CLCs) can also undergo structural changes through photoactivation, enabling control over the helical axis. Li et al. utilized light to manipulate the helical axis of CLCs in three dimensions, including altering its direction and inducing chiral inversion.^[^ ^135^ ^]^ The authors discovered that by applying light stimulation, the spiral axis can be reversibly transformed from vertical to parallel, followed by planar rotation on the substrate surface. This study provides valuable insights and references for the design and application of photo‐switchable molecules like diarylethylene. The switching effect of diarylethene molecules embedded between gold electrodes can be measured in situ by controlling light radiation, allowing the characterization of molecular junction conductivity in open and closed states. Research studies have demonstrated that single molecules can undergo ring‐forming isomerization when exposed to light.^[^ ^136^, ^137^ ^]^

Previous research focused on single diarylene change, achieving reversible photoswitching in devices composed of a diarylethene monolayer exposed to ultraviolet light (UV) or visible light.^[^ ^127^ ^]^ To investigate this reversibility, diarylethene molecules with ethylene anchor groups were self‐assembled on silicon substrates and irradiated with ultraviolet (313 nm) and visible light (578 nm) for 0.5h and 2h.^[^ ^110^ ^]^ This enabled reversible switching between closed‐loop and open‐loop states, and the conductance of the molecules in these states was measured using CP‐AFM. Such work elucidates the relationship between molecular structures and switching properties, offering a robust design principle for optoelectronic devices. Other relevant research has also been reported.^[^ ^26^ ^]^ Device fabrication involves depositing a thin layer of diarylethene oligomers (oligo(DAE)) on glassy carbon and gold electrodes via electrochemical reduction of a diazonium salt. The AFM tip serves as the top electrode, with DAE molecular layer thicknesses fixed at 2–3 nm and 8–9 nm, corresponding to different transport regimes. Both layers can be reversibly switched, exhibiting low ON/OFF ratios (2–3) in the direct tunneling regime and reproducible ON/OFF ratios above 200 in the hopping regime.

**Figure 2:**
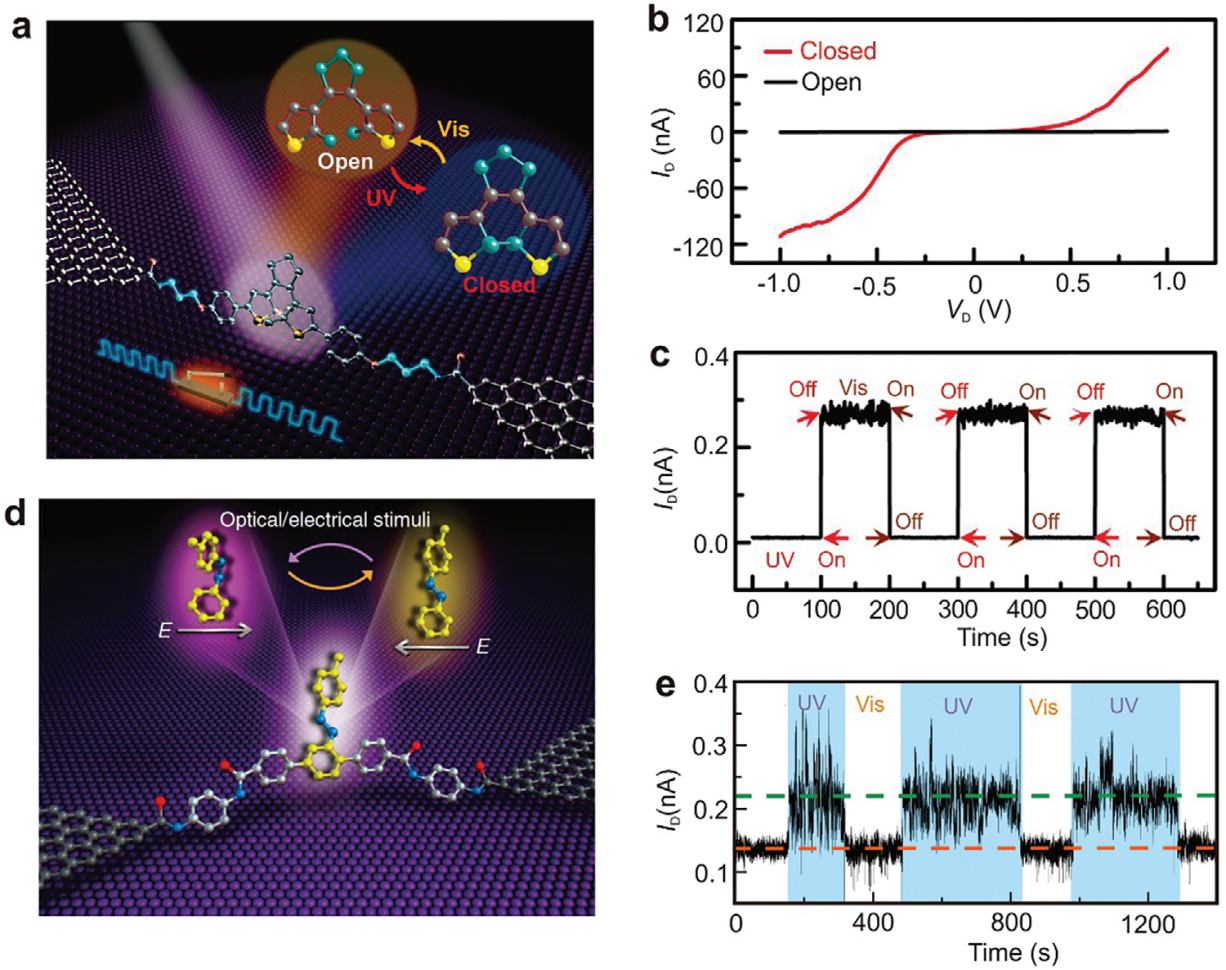
Photoisomerized single molecule switch. a) Schematic of the graphene‐diarylethene‐graphene junction that emphasizes the molecular bridge with extended methylene groups. b) *I‐V* characteristics of individual diarylethenes in open (black line) and closed (red line) form at gate voltage *V*_G_ = 0 V. c) Real‐time measurement of the electric current through a diarylethene molecule that reversibly switches between the closed and open forms respectively. a‐c) Reprinted with permission from ref. [2] Copyright 2016, AAAAS. d) Schematic representation of the device structure that highlights a reversible isomerization of the azobenzene unit between *trans* and *cis* forms triggered by either optical or electrical stimuli. The azobenzene side group serves as a chemical gate to modulate the conductance of the molecular backbone. e) Real‐time measurement of the electric current through an azobenzene molecule that reversibly switches between the *trans* and *cis* forms respectively. d, e) Reprinted with permission from ref. [144] Copyright 2016, Springer Nature.

Diarylene‐based monomolecular switching devices have gained significant attention due to their unique properties. Several key reports highlighted the importance of molecule‐electrode coupling strength in determining the performance of these devices. Initially, Au‐S bonds were used to prepare diarylene monomolecular devices. Unidirectional switching from the closed to open form was observed, and the reversed switching from the open to closed form is absent due to the gold electrode quenching the molecular excited state.^[^ ^138^ ^]^ In contrast, graphene electrode‐based single molecular switching devices exhibited a phenomenon where the molecule remained locked in its closed form, owing to energy transfer from the photoexcited molecule to the electrode's extended *p*‐electron system.^[^ ^91^ ^]^ This effect stemmed from strong molecule‐electrode couplings facilitated by equivalent amide links, which enhanced device stability.^[^ ^91^ ^]^ To address this issue, researchers incorporated three methylene (CH_2_) groups into each side of the molecular backbone, reducing the effective molecule‐electrode coupling, as shown in **Figure** **2**a.^[^ ^2^ ^]^ Consequently, as depicted in Figure 2b, the switch ratio of the source current reached approximately 100 under corresponding switch states with a gate voltage of 0 V and a source voltage of ±1 V. Figure 2c shows that after exposing single‐molecule switching devices to ultraviolet (UV) and visible light (Vis) radiation, the current of the diarylethylene molecule was measured in real‐time, and the molecule reversibly switched between closed and open forms.

Furthermore, studies have demonstrated that the conjugation strength of molecular side chains significantly affects the switching state transition, particularly the switching ratio.^[^ ^131^ ^]^ Molecules with conjugated side chains typically respond to visible light at wavelengths ≈570–630 nm, while those with nonconjugated side chains are triggered at 460 nm. This distinction arises from the spatial extent of the π‐electron system, which is smallest for molecules with nonconjugated side chains. It has been observed that open forms exhibit higher excitation energies, indicating larger HOMO‐LUMO gaps than closed forms, and that both open and closed forms have larger gaps than their counterparts with conjugated side chains. An analysis of *I‐V* curves within the single‐level transport model revealed variations in the alignment of the dominant transport level and the level broadening between open and closed forms, with the π‐electron system's delocalization significantly amplifying the coupling.

### 3.3 Azobenzene Switches

Azobenzene derivatives have emerged as prominent candidates for optical switch molecular junctions due to their accessibility and chemical stability.^[^ ^108^, ^111^, ^112^, ^113^ ^]^ These photoisomeric molecules consist of two benzene rings linked by N = N double bonds, which can be reversibly isomerized between *trans* and *cis* conformations upon light irradiation (refer to Table 1 for the structure).^[^ ^114^, ^115^, ^139^, ^140^ ^]^ The *trans*‐form adopts a planar conformation, while the *cis*‐form features a curved conformation with both benzene rings twisted at ≈55° relative to each other. *Trans*‐to‐*cis* isomerization occurs under UV, corresponding to the energy gap of the π‐π* transition. At the same time, *cis*‐to‐*trans* conversion is normally induced by visible light, matching the energy gap of the n‐π* transition.^[^ ^141^, ^142^ ^]^

However, using UV light for photoisomerization in most azobenzene‐based photoswitches presents limitations in biological systems, where UV light may elicit undesired reactions, such as cellular apoptosis. Researchers have discovered that substituting all four ortho positions with methoxy groups in an amido azobenzene derivative results in a significant red shift of ≈35 nm of the *trans* n‐π* band, effectively separating it from the cis n‐π* transition.^[^ ^143^ ^]^ Consequently, *trans*‐to‐*cis* photoswitching can be achieved using green light (530–560 nm), while reverse (*cis*‐to‐*trans*) photoswitching can be performed with blue light (460 nm). This approach enables bidirectional photoswitching between thermally stable isomers without the need of UV light, enhancing the compatibility of azobenzene molecules with biomedical research and broadening the application scope of photoisomeric molecules.

In the following paragraphs, we will discuss the development of azobenzene‐based single‐molecule optical switching devices focusing on chemically gated and fully reversible single‐molecule switches. This switch, which can be looked at as a chemically gaged transistor, employs a robust graphene‐molecule‐graphene single‐molecule junction (GMG‐SMJ) and incorporates an azobenzene unit covalently sandwiched at the side position of a terphenyl aromatic chain between graphene point contacts, as illustrated in Figure 2d.^[^ ^144^ ^]^ The switch exhibits two distinct modes: asymmetric stochastic conductance switching resulting from electric field‐induced isomerization of azobenzene and reversible photoswitching at low bias. A key finding is that both quantum mechanical calculations and experimental data demonstrate that an electric field can explicitly modulate the energy difference between the *trans* and *cis* forms of azobenzene, as well as the energy barrier for conformational changes. This evidence suggests that electric fields can be employed to adjust the energy alignment and transition energy barrier of *trans* and *cis* forms, enabling isomerization of the substituted azobenzene unit within a single‐molecule electrical circuit.

Meanwhile, real‐time measurements exhibit reversible switching between *cis* and *trans* forms upon exposure to UV and visible light irradiations, as depicted in Figure 2e. The device's conductance can be reversibly switched between a high state and a low state, with an on/off ratio of ≈2.1, while applying a minimal positive voltage of 0.01 V to reduce the influence of the electric field. Control experiments employing a device reconnected by a terphenyl aromatic molecule without the substituted azobenzene side group did not display similar photoswitching behavior, confirming that the photoswitching effect and electric field‐induced stochastic switching originates from the reversible trans‐cis isomerization of azobenzene. These devices function as chemically‐gated, fully‐reversible, single‐molecule molecular circuits, offering new insights for the development of practical single‐molecule devices and logic gates.

Reversible conductance switches under UV and visible light irradiation have been achieved by employing CP‐AFM technology in ultra‐high vacuum to create azobenzene molecular devices with various bottom electrodes.^[^ ^145^ ^]^ A theoretical study highlights the direct correlation between strong or weak conductance states and the molecular configuration in electron transport through gold‐azobenzene‐gold (Au‐AZP‐Au) junctions.^[^ ^146^ ^]^ The research employs the non‐equilibrium Green's function formalism and density functional theory (DFT) to elucidate these phenomena. The findings reveal that Au‐AZP‐Au junctions are promising candidates for light‐driven molecular switches without additional functionalization processes. This insight contributes to the understanding and development of azobenzene‐based single‐molecule optical switching devices in the field of molecular electronics.

Although the reversible *trans*→*cis* isomerization of azobenzene derivatives in free space induced by light irradiation has undergone extensive investigation, which isomers embedded in the circuit will own higher conductivity is still in debate. Theoretically, the non‐parallel orientation of the two benzene rings in the *cis* isomer, disrupting the conjugation of their π system, predicts that the single‐molecule junction of the *cis* isomer generally exhibits lower conductivity compared to the *trans* isomer.^[^ ^147^, ^148^, ^149^ ^]^ In contrast to these predictions, the majority of experiments indicate that *cis* isomers typically manifest a higher electrical conductivity than *trans* isomers.^[^ ^150^, ^151^, ^152^, ^153^ ^]^ This higher conductivity is mainly attributed to the existence of shorter transport path, enhanced delocalization of frontier orbital, or special electronic state for *cis*‐ isomer based molecular junctions. Consequently, a comprehensive investigation of the charge transport through the azobenzene derivatives with different anchoring groups is necessary for future exploration. Recently, Tan et al. demonstrated that the most probable conductance of amine‐anchored azobenzene‐based molecular junctions increases continuously upon UV irradiation.^[^ ^154^ ^]^ In contrast, the conductance of pyridyl‐anchored molecular junctions with identical azobenzene cores exhibits an opposite trend, highlighting that the anchoring groups play a pivotal role that potentially overrides (even reverses) the effects of photoinduced conformational changes.

Similar to azobenzene, Li and co‐workers designed a series of molecules containing a halogen bond donor and incorporated it into a self‐assembled soft helix superstructure.^[^ ^155^, ^156^ ^]^ Through the controlled irradiation of visible light, this molecular switch exhibited reversible unfolding of helical structures and chiral inversion. This study highlights the potential application of visible light‐driven molecular switches in self‐assembled soft spiral superstructures, offering novel ideas and methods for the development of innovative visible light‐responsive materials.

### 3.4 Other Photoisomeric Switches

In addition to the photochromic molecular switches discussed previously, numerous alternative candidate molecules exhibit promising potential for optical switching applications.^[^ ^122^, ^123^, ^157^, ^158^, ^159^, ^160^, ^161^, ^162^ ^]^ Among these, spirogyra molecules are of particular interest, comprising an indoline and a chromene moiety linked by a spiro, with the two moieties oriented perpendicularly to one another, as shown in Table 1.^[^ ^116^, ^117^ ^]^ The optical spectrum of the closed‐ring isomer displays two distinct localized transitions: a band at 272–296 nm corresponding to the π–π* electronic transition in the indoline section, and another at 323–351 nm associated with the chromene moiety.^[^ ^118^ ^]^

The complex structure of spiropyran molecules allows for a variety of stimulation modes to activate switching, including solvents, metal ions, acids, bases, temperature, redox potential, and mechanical force. Recent advancements have led to the development of spiropyran‐based molecular switching devices that respond to both optical and chemical stimuli through electrical conductivity changes.^[^ ^119^ ^]^ By utilizing a light pointer or chemical signal, the isomerization of bifunctional spiropyran derivatives can be rapidly and reversibly induced in a bulk reservoir, resulting in a switch between low and high electrical conductivity levels. To ensure practical response times, the spiropyran derivatives are chemically functionalized for rapid isomerization. The remarkable multi‐stimuli responsiveness and synthetic versatility of spiropyran derivatives in controlling switching mechanisms make them excellent candidates for future molecular circuitry applications.

Another application of spiropyrane molecules is using it as photosensitive molecules/groups. Li et al. designed a molecule containing photosensitive groups and incorporated it into self‐assembled supramolecular structures.^[^ ^163^ ^]^ By illuminating this structure with light, they observed morphological changes and the modulation of dissipative properties. This photoactivated deformation effect is achieved through the photochemical reactions of photosensitive groups, which leads to the rearrangement of supramolecular structures and morphological changes. This study provides new insights and methods for designing and preparing smart materials with controllable morphological changes and dissipative properties. This group also successfully constructed a light‐fueled dissipative supramolecular self‐assembly system in water.^[^ ^164^ ^]^ By irradiating this assembly system with light, they observed changes in fluorescence. Through this approach, they successfully achieved modulation of fluorescence by the supramolecular assembly system formed in water. This study demonstrates the potential of a photosensitive supramolecular assembly system implemented using light‐driven methods as a fluorescence modulator in water, offering new ideas and methods for designing and preparing smart materials with tunable fluorescence properties.

Dimethyldihydropyrene (DHP) has emerged as a versatile molecular platform for the design of optoelectronic materials and molecular devices due to its chemical functionalization capabilities.^[^ ^120^, ^165^ ^]^ Despite its complex synthesis routes in comparison to other optical switch molecules,^[^ ^121^, ^166^ ^]^ DHP derivatives have shown significant potential as addressable nanoscale molecular building blocks. Studies have demonstrated the reversible photothermal conversion of dimethyl dihydropyrene/cyclopentane double electrochromic systems with dipyridine through UV‐visible spectroscopy, nuclear magnetic resonance, and electrochemical measurements.^[^ ^120^ ^]^

Dihydroazulene (DHA) and vinylheptafulvene (VHF) based photochromic systems are another family of molecular switches that are garnering interest in advanced materials and molecular electronics research.^[^ ^158^, ^159^ ^]^ DHA converts to VHF when exposure converts to VHF when exposed to ultraviolet light, facilitated by a 10‐electron retro‐electrocyclization reaction,^[^ ^160^, ^161^ ^]^ and exists in equilibrium between two distinct geometric and conductive states. VHF reverts to DHA through a thermally activated ring‐closing reaction upon exposure to heat.^[^ ^157^, ^162^ ^]^ Compared to the *trans*‐to‐*cis* isomerization of azobenzenes, DHA's photoreaction to VHF derivatives yields higher quantum efficiencies. Furthermore, the absorption maximum of DHA can be tailored by modifying the substituents on its functional moiety.^[^ ^122^, ^123^ ^]^

Another significant class of photo‐switchable molecules is cyanodiarylethene, which can exhibit distinct light‐triggered changes in their emission colors. α‐Cyanodiarylethenes, that is, α‐cyano‐functionalized diarylethenes, as a photochromic molecular switches, are extensively investigated due to their unique characteristics (*e.g*., their aggregation‐induced emission or aggregation induced enhanced emission behavior in their self‐assembled states, and visible changes in fluorescence colors during the photoisomerization). Li et al. provided a comprehensive overview of the design, synthesis, properties, and applications of fluorescent photochromic α‐cyanodiarylethene molecular switches.^[^ ^167^ ^]^ This work serves as a valuable reference and guideline for the investigation of these molecules, offering novel ideas and methodologies for further research in related fields.

Theoretical calculations have confirmed that the conformational change in benzoquinone derivatives results in significant changes in single‐molecule conductivity by several orders of magnitude.^[^ ^168^ ^]^ This change is attributed to the reversible crossover from destructive interference in the *trans* isomer to constructive interference in the *cis* isomer, leading to a substantial conductance change at room temperature (>1000 times). Collectively, these investigations demonstrate the prospective utilization of DHP derivatives and DHA/VHF photochromic systems as molecular devices for optical switching, offering a viable substitute for conventional single‐molecule switches, namely diaryls and azobenzenes. Such sophisticated materials offer novel avenues for the advancement of high‐performance optoelectronic devices and molecular electronics.

## 4 Charge Controlled Molecular Switches

Molecular switches exhibit a wide range of behaviors, with redox‐active molecules being a prominent example. These molecules demonstrate switching behavior primarily due to alterations in their charge state in response to external stimuli, such as electrochemical reactions, charge transfer, or electron induction. This type of switching is referred to as charge‐controlled switching. To facilitate a structural understanding, we have categorized charge‐controlled molecular switches into three broad classifications based on the different aspects of the redox process: electrochemical gating, charge transfer, and electron induction.^[^ ^169^ ^]^

### 4.1 Molecules with Redox Properties

Redox‐active molecules play a critical role in oxidation‐reduction reactions by facilitating electron transfer. The fundamental mechanism underlying oxidation‐reduction reactions involves the transformation of a substance's structure through electron transfer. In these reactions, one substance loses electrons (undergoes oxidation) while another gains electrons (undergoes reduction). The electron transfer process occurs through a series of chemical reactions. Molecules exhibiting redox properties typically possess the following structural features:

These molecules should have stable electron structures and appropriate electronegativity to ensure seamless electron transfer between the substances. As they exhibit significant conductance changes between their two states, redox‐active molecules become ideal candidates for conductance switches. Several types of molecules can be considered, including redox‐active organic molecules, viologens,^[^ ^170^ ^]^ oligoanilines,^[^ ^171^ ^]^ oligothiophenes,^[^ ^172^ ^]^ ferrocene,^[^ ^173^ ^]^ anthraquinone,^[^ ^174^, ^175^ ^]^ benzodifuran,^[^ ^176^ ^]^ pyrrole‐tetrathiafulvalene (TTF),^[^ ^177^ ^]^ unsubstituted and substituted oligo (phenylene ethynylenes) (OPEs),^[^ ^178^, ^179^ ^]^ perylene tetracarboxylic bisimide (PBI),^[^ ^9^, ^180^ ^]^ pyrrolidine‐substituted perylene tetracarboxylic diimide (PTCDI)^[^ ^181^ ^]^ redox‐active proteins,^[^ ^182^ ^]^ and transition metal zzomplex.^[^ ^183^ ^]^ **Table** **2** shows the structures of several typical redox‐active molecules. Metal‐ligand compounds undergo redox mechanisms that involve charge transfer between the metal and ligands and the breaking and formation of coordination bonds. In contrast, organic molecules such as anthraquinone derivatives experience electron transfer between functional groups like hydroxyl groups and benzene rings during redox reactions. The specific redox mechanisms vary for different compounds and depend on factors like metal and ligand properties or functional groups involved.

**Table 2:** Several typical redox‐active molecules.

Metal ligand‐based molecules	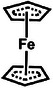	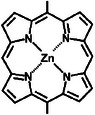	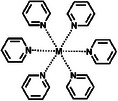	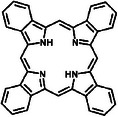
Other redox molecules	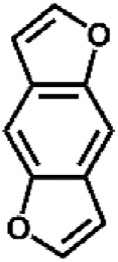	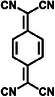	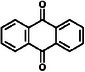	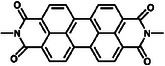

### 4.2 Electrochemical Gated Switches

Electrochemical gating is a technique that enables precise control of the voltage difference between two working electrodes, specifically the source and drain, as well as the potential drop between each working electrode and a reference electrode at the interface between a charged solid and liquid. In this method, the electrolyte serves as a gate, exhibiting a strong coupling with the externally applied electric field. The fundamental principle underlying electrochemical gating is the accurate adjustment of the Fermi level of the electrode, which alters the redox state of the molecular functional unit, enabling the switching function with high precision. By accurately manipulating the Fermi level of the electrode, it is possible to control the electrochemical properties of the molecules, such as conductivity, which can be applied in various fields including sensing, data storage, and electronic devices.

The concept of an “electrolyte gate” for regulating charge transport in molecular electronics was initially proposed by Wrighton and coworkers, Meulenkamp et al.,^[^ ^184^, ^185^ ^]^ and further developed by Tao and Venkataraman et al.^[^ ^186^, ^187^, ^188^ ^]^ The electrochemically controlled STM‐BJ technique was employed to investigate charge transport through core‐substituted naphthalene diimide (NDI) molecule junctions, and the results were compared with simulations based on DFT calculations.^[^ ^189^ ^]^ NDI has attracted significant attention in organic electronics and supramolecular chemistry due to its electron acceptor properties and n‐type semiconductor characteristics. The ability to substitute the naphthalene core has sparked interest in exploring charge transport through the naphthalene unit. The diimide unit serves as a functional pendant with robust coupling to the naphthalene backbone, and it is sensitive to external stimuli such as applied electrochemical gating.

**Figure 3:**
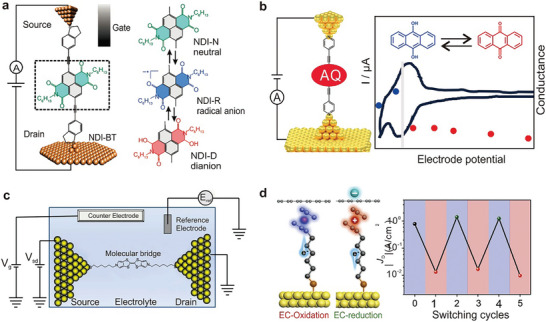
Several electrochemically regulated single molecule switches. a) Schematic illustration of the electrochemically gated break junction experiment and molecular structure of NDI‐BT in the neutral state (NDI‐N), radical‐anion state (NDI‐R), and dianion state (NDI‐D). Reprinted with permission from ref. [189] Copyright 2015, Wiley‐VCH GmbH. b) Construction of electrochemically gated AQ single‐molecule switch device based on STM‐BJ technology, in which the high and low conductance correspond to different redox states. Reprinted with permission from ref. [19] Copyright 2014, American Chemical Society. c) An electrochemical molecular break junction with electrolyte gating based on four electrodes. Reprinted with permission from ref. [193] Copyright 2016, American Chemical Society. d) Schematic illustration of EC oxidation and EC reduction with controlled electron transfer processes and corresponding switching. Reprinted with permission from ref. [194] Copyright 2020, Cell.

By applying an appropriate potential, two sequential electron transfer reactions can transpire, transforming the neutral species (NDI‐N) into the radical anion (NDI‐R) and ultimately into the dianion species (NDI‐D). This property renders the core‐substituted NDI molecule an exemplary molecular junction for evaluating the influence of pendant redox groups on single‐molecule conductance. As depicted in **Figure** **3**a, the NDI‐BT molecule can be switched between various charge states utilizing electrochemical gating, such as from its neutral state (NDI‐N) to its dianion state (NDI‐D) or between any two charge states. All three charge states are reversible and can be accessed by modulating the potential applied to the molecule. Notably, the molecule exhibits tristable charge states. While bistable states are more common, this distinctive tristable behavior distinguishes it from traditional on/off switches.

The reversible conductance adjustment of two distinct organic molecules containing anthraquinone (AQ) centers is achieved through electrochemical gating. The gate voltage regulates the AQ active molecules in their oxidation and reduction states, resulting in a switching ratio exceeding one order of magnitude, as illustrated in Figure 3b.^[^ ^19^ ^]^ Figure 3b presents the device structure diagram (left) and a graph of conductivity changes (right). The molecules employed in this study feature AQ groups, which undergo oxidation‐reduction reactions when the applied potential reaches the oxidation‐reduction potential of the AQ molecules. Electrochemical cyclic voltammetry is utilized to apply potential, thus generating isomers that cause significant conductivity alterations, as demonstrated in the right panel of Figure 2b.^[^ ^19^ ^]^ Gating in this work induces a moderate change in conductivity by shifting the Fermi level relative to molecular resonance. At the oxidation‐reduction potential, a substantial and reversible conductivity jump is observed due to the change in the oxidation‐reduction state, accompanied by a transition in the conjugation pattern from linear (reduction state) to cross conjugation (oxidation state).

A high‐performance single‐molecule switch was identified through first‐principles‐based calculations, which examined the intramolecular proton transfer's role in altering the electron wave function's parity.^[^ ^190^ ^]^ This switch was investigated in molecular junctions, comprising either a keto or enol tautomer placed between two magnetic zigzag graphene nanoribbon (GNR) electrodes. The study assessed the impact of intramolecular proton transfer on the current, revealing significant switching properties with an on/off ratio as high as 3.4 × 10^2^.^[^ ^190^ ^]^ Furthermore, the application of an electrochemical gate voltage can also control the biological macromolecule DNA's conductance.^[^ ^191^ ^]^ By attaching a redox group to a DNA base and applying an electrochemical gate voltage to a single DNA molecule using STM, the DNA's conductance can be regulated. The electrochemical gate, a silver electrode, is employed to control the redox state of AQ‐DNA, where AQ substitutes one of the standard DNA bases for switching the DNA conductance. The authors demonstrated that, by using DNA as a probe, it is feasible to investigate the kinetics and thermodynamics of redox reactions at the single molecular level by observing the individual conductance switching. According to theoretical calculations, the conductance switch of DNA is due to the alteration in energy level alignment of the redox states relative to the Fermi level of the electrodes.

Besides electrochemical gating with traditional electrolyte solutions, the electrical properties of single‐molecule junctions (such as viologen molecular bridges) have also been investigated in aqueous electrolytes using ionic liquid gates.^[^ ^192^ ^]^ By employing an electrochemical four‐electrode method, the conductance of the single molecular junction can be successfully adjusted by altering the gate voltage. In this setup, the STM tip and the gold substrate serve as source and drain electrodes, respectively, while counter and reference electrodes complete the electrochemical system. The viologen molecule acts as a molecular bridge connecting the source and drain electrodes. This investigation highlights the advantages of using ionic liquids as a gating medium over aqueous media or solid‐state three‐terminal platforms fabricated through nanolithography.^[^ ^192^ ^]^ Electrochemical reduction can increase single‐molecule conductivity, which can be controlled through liquid electrochemical gating. This concept is referred to as the “bipyridinium (or viologen) single‐molecule transistor configuration,” where the gate voltage is provided by the controllable potential achieved through the electrochemical double layer. Ionic liquids offer a more efficient gate coupling compared to aqueous electrolytes. Furthermore, by encapsulating the bipyridinium redox moiety within a molecular cage, the surrounding environment can be controlled, thereby modulating molecular conductivity.^[^ ^193^ ^]^

Figure 3c illustrates the approach for measuring the conductance of single molecules in an electrochemical environment. The molecular bridge connects two metallic electrodes facing each other and is separated by nanometer dimensions. The attachment is facilitated by chemisorption groups at both ends of the molecule, enabling the measurement of the electrical properties of the molecular junction. Additional electrodes are required to control the electrode potential. The contacting electrodes function as working electrodes 1 and 2, while the counter and reference electrodes are placed at a distance from the nanogap. This configuration, known as a four‐electrode bipotential static setup, allows for independent potential control of the two working electrodes, a method commonly used with in situ electrochemical STM. Consequently, the electrode potential can be altered without changing the bias voltage between the contacting electrodes. In Figure 3c, the two contacting electrodes are labeled as source and drain, while a “gating voltage” (V_G_) is generated by the combination of counter electrode/reference electrode. As a result, the molecule is placed in an arrangement equivalent to a “single molecule transistor configuration”, where the gate voltage is provided by the controllable potential achieved through the electrochemical double layer.

Apart from single molecular switches,^[^ ^193^ ^]^ mono‐layer molecular switches are also reported, in which a ferrocene monolayer is introduced on a gold electrode as a molecular switch. The switch functionality is realized through the redox properties of ferrocene and the employment of a single layer of graphene (SLG) as the top electrode.^[^ ^194^ ^]^ By applying an electrochemical potential, the redox reaction of the ferrocene group beneath the graphene is initiated, resulting in a change in the distance between the top graphene electrode and the Fc‐SAM contact, as illustrated in Figure 3d. This alteration leads to a transition between a high‐conductance neutral state with an asymmetric *J*_D_‐*V*_D_ curve and a low‐conductance oxidation state with a symmetric *J*_D_‐*V*_D_ curve. An on/off ratio exceeding two orders of magnitude is achieved. During these redox reactions, charges traverse the single‐layer graphene. The electronic transparency and ion impermeability of graphene enables counter anions in the oxidation state to remain separated on the upper surface of the graphene layer, counterbalancing the oxidized Fc cations underneath the graphene. This mechanism allows for electrochemical control of cross‐planar charge transport in the Au/Fc‐SAM/SLG junctions.

A recent study on electrochemical gating switches demonstrates a method wherein the pyridine nitrogen of the test molecule interacts with a cationic reagent, forming pyridine and modifying the molecule's frontier orbitals, leading to changes in conductivity. This modification facilitates switching, allowing the on/off switch to function.^[^ ^195^ ^]^ Another recent investigation showcases the use of electrochemical gating to control the interaction between active redox molecules and counterions in a solution, enabling the transition between high and low conductivity states.^[^ ^196^ ^]^ The switching ratio and the applied gate voltage were found to be dependent on the counterion species used in the solution. This innovative approach draws attention to the critical role played by counterion species in electrochemical charge transport in redox‐active molecules. The findings of this study have significant implications for the development of novel energy storage systems and programmable molecular electronic devices.^[^ ^196^ ^]^

### 4.3 Charge Transfer‐Induced Switches

Redox molecules possess an inherent ability to oscillate between oxidation and reduction states through the gain and loss of electrons. This unique property enables them to exhibit charge transfer behaviors in response to external stimuli, such as the injection of a charge that remains within the molecule and interacts with it. Recent advances in the field of charge transfer‐induced molecular switches have led to novel developments, including the use of a molecular layer of phthalocyanine adsorbed on the surface of a metal electrode. This arrangement facilitates bistable switching on the surface to manipulate tin ions. The vertical displacement of the central tin ion within the phthalocyanine molecular framework can be achieved by injecting resonant electrons or holes into the molecular orbital. This process induces a change in the charge distribution within the molecule, resulting in the movement of the tin ion, which can be either upward or downward, depending on the direction of charge injection. Based on these principles, a single‐molecule switch has been realized for the first time in an ordered layered array structure. This achievement demonstrates that a tightly packed SnPc layer can effectively decouple the second molecule layer from the metal surface, enabling localized and reversible conversion.^[^ ^197^, ^198^, ^199^ ^]^

A reversible molecular switch that leverages the bonding between gold atoms and perylene‐3,4,9,10‐tetracarboxylic dianhydride (PTCDA) molecules.^[^ ^200^ ^]^ This switch is implemented on a thin NaCl film supported on a copper substrate by using a self‐assembled composite STM/AFM system. The switch can be toggled between bonded and non‐bonded states by applying a bias. These two states exhibit distinct charge configurations, resulting in a notable change in tunneling current that reflects the switch characteristics. The switch performance can be inferred from the on/off ratio of the tunneling current, which is roughly two orders of magnitude. The exceptional reliability and stability of this switch are attributed to the extended lifespan of electrons or holes bound to the Au‐PTCDA complex, allowing the complex to overcome energy barriers between connected and non‐bonded configurations on the excited‐state potential energy surface.

**Figure 4:**
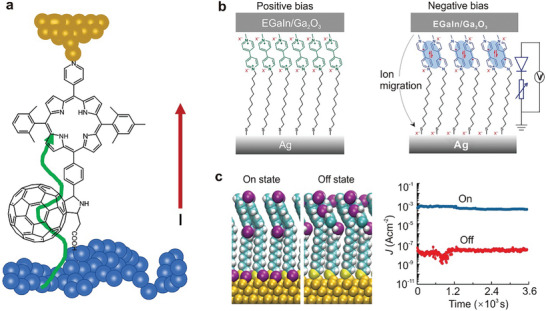
Several charge transfer‐induced switches. a) The expected bonding geometry of the porphyrin‐C_60_ dyad molecule in the gold‐ITO tunnel junction. A carboxylate interacts with ITO and a pyridyl moiety binds to the gold probe. Reprinted with permission from ref. [93] Copyright 2011, American Chemical Society. b) Schematic illustration of Ag‐S (CH_2_ MV^2+^ or PF_6_^−^) at positive and negative bias. c) Periodic DFT calculations were performed on [MV^•+^]_2_ SAM (open state) and MV^2+^ SAM (closed state) on Au (with purple counterions, mint green carbon, white hydrogen, ocher gold, and yellow sulfur), and current density corresponding to the on and off state. b, c) Reprinted with permission from ref. [202] Copyright 2020, Springer Nature.

An alternative switch design, based on a porphyrin‐fullerene dyad molecule, is created using STM technology. This molecular switch forms a charge‐separated state when exposed to light, as illustrated in **Figure** **4**a.^[^ ^201^ ^]^ Upon illumination, a considerable portion of the molecules become more conductive, reverting to a lower conductance in the dark. Light absorption triggers the formation of P^•+^‐C_60_^•−^ through photoinduced electron transfer and causes the charges to migrate away from the site of initial electron transfer. This migration occurs via hopping to adjacent molecules and/or movement into the ITO conductive substrate. The slow recombination of these separated charges allows the charge‐separated state to be observed in conductance measurements. The unexpected interaction with the ITO substrate offers novel opportunities for charge extraction from photoexcited dyads, potentially paving the way for the development of advanced molecular photovoltaic devices.

On the other side, the MCBJ technique is used to investigate charge‐carrier transport in Au‐bipyridyl‐dinitro oligophenylene‐ethynylene dithiol (BPDN‐DT) molecule‐Au junctions.^[^ ^84^ ^]^ The investigation reveals that these junctions exhibit reversible switching behavior between different conductance states, dependent on the applied voltage. The molecular junction can easily switch between “on” and “off” states under different voltage conditions. This behavior in the BPDN‐DT system may ascribed to a conformational change induced by the nitro group, which could result from the trapping or tilting of charges on the molecule. These insights contribute to our understanding of charge transport mechanisms in molecular junctions and hold promise for the advancement of future electronic devices

Aiming to elucidate the mechanisms underlying conductance hysteresis and switching behavior, the researchers create magnesium porphine (MgP) molecular junctions and examine the interfacial charge transfer.^[^ ^201^ ^]^ They characterized the junction behavior at the atomic scale by using the tip of STM at a low temperature of 10 K to create a single‐molecule double‐barrier tunnel junction. They confirmed that hysteresis and switching behavior originated from the single‐molecule junction itself by investigating the bare oxide surface adjacent to the molecule and found no evidence of these phenomena. Certain charge states showed additional stability, which led the researchers to conclude that charge bistability was responsible for the hysteresis phenomena. Also, they recognized that the configurations of molecules adsorbed on the gold surface played a crucial role in determining the relative strength of different charge states. This study offers critical insights into the mechanisms underlying conductance hysteresis and switching behavior in molecular junctions.

Although most reports on molecular devices focused on single‐function devices, recent research has begun to shift toward multi‐functional systems that demonstrate the potential for more complex and versatile applications. In a recent study, researchers used an Ag bottom electrode, a GaOx/EGaIn top electrode, and a SAM of S(CH_2_)_11_MV^2+^X^−2^ to prepare molecular devices with an Ag‐SAM‐EGaIn/GaOx sandwich structure.^[^ ^202^ ^]^ In this configuration, the electric field drives the migration of ions, resulting in dual functionality characterized by large resistive on/off and rectification ratios. The interaction processes occurring in molecular junctions involve not only the molecule and the electrodes but also interactions within the molecule itself. Figure 4b presents a schematic of the junctions, where MV is methyl viologen and X^−^ is the counterion. MV has three oxidation states, and MV^•+^ dimerizes to form a stable dimer complex driven by π–π stacking and pairing of the electron spins of each MV^•+^. In this system, the diode rectifies the current with a high rectification ratio, achieved by the internal asymmetric placement of the MV^2+^ cells at the junction. The diode is in series with a variable resistor, and the formation of [MV^•+^]_2_ provides a high on/off ratio between the high and low conductance states of the junction. Figure 4c shows electron structure calculations performed on isolated dimers and SAM. The calculated complexes confirm the formation of tightly bound complexes by [MV^•+^]_2_ with dimerization energy driven by π‐π interactions and electron spin pairing. Upon dimerization, the HOMO‐LUMO gap of all halides is significantly reduced, which partially explains the increase in conductivity upon dimerization. The study also revealed the influence of counterion size on the reversibility of dimerization and ion migration, pointing to a two‐step switching mechanism involving charging, followed by dimerization and ion migration.

The switching effect observed in self‐assembled methyl viologen molecular layers can be attributed to the longer alkyl chains, which serve to separate the LUMO from the bottom electrode, enabling coupling with the top electrode. Under negative bias, the LUMO energy level of the molecule enters the bias window, allowing electrons to be injected into the top electrode. Consequently, the radical cations MV^•+^ are formed, increasing the device conductance as excess counter ions migrate to the bottom electrode. Notably, the HOMO–LUMO gap of [MV^•+^]_2_ is smaller than that of MV^2+^, allowing the HOMO of [MV^•+^]_2_ to enter the conduction window at negative bias, increasing the conductance of the junction significantly. This discovery has far‐reaching implications, as it can be extended to other redox systems involving (supramolecular) chemical reactions, substantial conformational changes, or dimerization. As such, it holds considerable development potential and a broad application scope in the field of molecular switching devices. Notably, this study primarily focuses on performance in supramolecular systems with large‐area junctions rather than single‐molecule junctions. Some of the best performing molecular switches are in large‐area junctions.

Large‐area junctions differ from single‐molecule devices in that they can be produced on a larger scale, thus offering higher scalability and practicality. Bert de Boer et al.^[^ ^203^ ^]^ demonstrated a cross‐bar method for manufacturing molecular junctions with a contact diameter of up to 100 µm, achieving high yields (>95%). The molecular structure displayed excellent stability and reproducibility. This straightforward method holds promise for cost‐effectiveness and could pave the way for practical molecular electronics. In recent years, notable advancements have been made in the field of molecular electronics with large‐area junctions, and more detailed advancements for molecular switches with large‐area junctions can be found somewhere else.^[^ ^54^, ^204^, ^205^, ^206^, ^207^, ^208^ ^]^

### 4.4 Current‐Induced Switches

**Figure 5:**
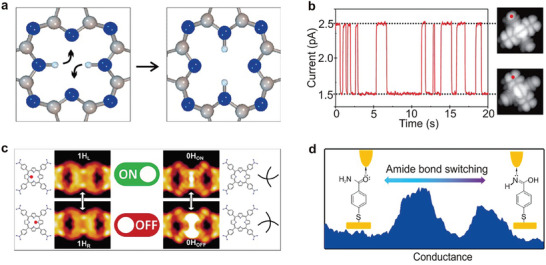
Several typical current‐induced switches. a) Schematic diagram of the hydrogen tautomerization reaction for the switching behavior. b) (Left) The current trajectory was obtained when the tip of STM was located at one end of the molecule. (Right) Orbital images showing the orientation of the LUMO corresponding to the high or low current state (red dot in STM images). a, b) Reproduced with permission from ref. [210] Copyright 2007, AAAAS. c) Binary H‐tautomerization in the central 1H‐TAPP and stereoisomeric conformational switch in two 0H‐TAPPs. Reprinted with permission from ref. [209] Copyright 2020, American Chemical Society. d) The formation of N‐C single and double bonds and the change of conductivity state due to proton transfer. Reproduced with permission from ref. [212] Copyright 2021, American Chemical Society.

Switching devices extend beyond the realm of “charge‐transfer switches” triggered by charge capture. Current can also stimulate energy and initiate molecular structural changes, resulting in a switching effect. Researchers have investigated the phenomenon of current‐induced hydrogen isomerization in phthalocyanine molecules.^[^ ^209^, ^210^ ^]^ As shown in **Figure** **5**a, according to DFT calculations, optical and the nuclear magnetic resonance (NMR) spectroscopy, this switching behavior can be attributed to the changes in the position of the imino hydrogens in the central cavity, specifically hydrogen tautomerization.^[^ ^210^ ^]^ The rotation of the entire molecule can be ruled out, as switching of molecules at step edges and in arrays of molecules is observed, where rotation is impossible. In Figure 5b, switching on an Xe monolayer is observed, where a 90° rotation of the molecule would be incompatible with the surface symmetry. By positioning the tip above the molecule and significantly increasing the bias above the LUMO resonance, hydrogen tautomerization can be induced. Lowering the bias and imaging the LUMO at resonance reveals two current levels corresponding to a 90° rotation of the LUMO orientation. The current signal or vertical‐tip function switches between two defined levels with a bias voltage of 1.7 V, emphasizing the role of electron and energy transport within the molecule.

As shown in Figure 5c, aminophenyl‐functionalized porphyrins (TAPPs) were shown to form weakly bonded chains on a gold (111) surface via hydrogen bonds between their amino‐terminations.^[^ ^209^ ^]^ The chemical and electronic structure of the TAPP chains is strongly influenced by the interplay between amino‐amino and amino‐gold substrate interactions. Using low‐temperature scanning tunneling microscopy (LT‐STM) and ab initio calculations based on DFT, the switching properties of each TAPP molecule along these chains were investigated. The chain formation is crucial for the switching properties of the molecules, which also depends on the protonation state of the inner macrocycle. Partially deprotonated donor TAPPs can undergo H‐tautomerization, while fully deprotonated TAPPs can undergo a binary stereoisomeric conformational switch. Using STM techniques, similar studies have also succeeded in achieving a four‐level conductance switch based on single proton transfer in porphyrin molecules.^[^ ^211^ ^]^

Additionally, employing STM‐BJ technology, the single‐molecule conductance of 4‐mercaptobenzamide (MBAm) was investigated, revealing a bimodal charge transport behavior of a single amide link.^[^ ^212^ ^]^ The difference in conductance was attributed to the distinct electron transfer pathways containing C═N and C═O double bonds. It is found that the C═N double bond significantly enhances the gold electronic overlap with the conjugated π‐backbone compared to the C═O double bond. The proton transfer from the N to the O atom was facilitated with the assistance of an adjacent water molecule bridge, leading to the formation of a C═N double bond and a significant enhancement of molecular conductance. The lone pairs of electrons on the oxygen atom could form an Au−O bond via coordination interaction when MBAm forms a single‐molecule junction in the electrode gap.

Azobenzene molecules can be utilized to construct molecular switches, which are activated by transmitting electrons above the threshold bias.^[^ ^213^ ^]^ To capture the isomerization process, high spatial resolution scanning tunneling spectroscopy (SR‐STS) measurements were conducted to map the electronic structures of both trans‐azobenzene and cis‐azobenzene, examining the charge distribution of a molecular state with high spatial resolution. The results indicate that the azobenzene molecule is a promising candidate for molecular switches. Specifically, the findings demonstrate that the trans‐azobenzene state can be reversibly transformed into the cis‐azobenzene state through three possible modes: local tunneling electron triggering, electron impact resonant excitation, or vibrational excitation. In addition to the azobenzene molecule, which can be initiated by a tunneling electron, there have been reports of inducing isomerization of single chloronitrobenzene molecules on a Cu(111) surface using STM at a temperature of 5K and verifying the isomerization reaction.^[^ ^214^ ^]^

## 5 Force‐Induced Molecular Switches

Force control is another fundamental approach for inducing switching characteristics in molecular/atomic switching devices. Force‐induced switching primarily relies on changes in molecular conformation, the evolution of the molecule‐electrode interface, and alterations in the molecular charge state to achieve the desired switching properties.

### 5.1 Molecular Structure Change Switches

Molecular conformation is one of the main factors affecting the device's performance. The various conductance states that arise from different isomers and active redox molecules, as well as structural changes in the molecule under the application of force, can significantly impact the device's conductance. Examples of such structural changes include the folding or twisting of the molecule,^[^ ^215^, ^216^ ^]^ and alterations in the dihedral angle when the molecule is subjected to stretching or compression.^[^ ^217^, ^218^, ^219^ ^]^ These changes can lead to considerable variations in conductance, enabling the device to switch between two conductance states.

Studies have reported the observation of a binary hysteresis effect in the conductance of a 1,4‐diethynylbenzene molecule, which was attributed to a mechanically induced *cis*‐*trans* conformational change.^[^ ^220^ ^]^ By forming robust covalent bonds between a molecule and silicon electrodes, the conformational modulation of the molecule can be stably and repeatedly controlled utilizing STM‐related techniques. Elongation of the molecular junction increases conductance, while compression decreases it. In this context, each terminal dihedral angle serves as a gate to control the switch of conductance, as it couples the electrode‐linker orbital into the σ framework.

Furthermore, it has been reported that the tunneling current increases with molecular elongation through mechanical means, as demonstrated with a cyclophane molecular model in the Green's Function Density Functional Tight‐Binding (GDFTB) computations.^[^ ^221^ ^]^ These simulation results suggest the potential of an electromechanical molecular switch based on transport‐extension behavior, which can be achieved through mechanical manipulation. The observed behavior occurs as molecular elongation opens up a more conductive channel for electron transport.^[^ ^221^ ^]^

**Figure 6:**
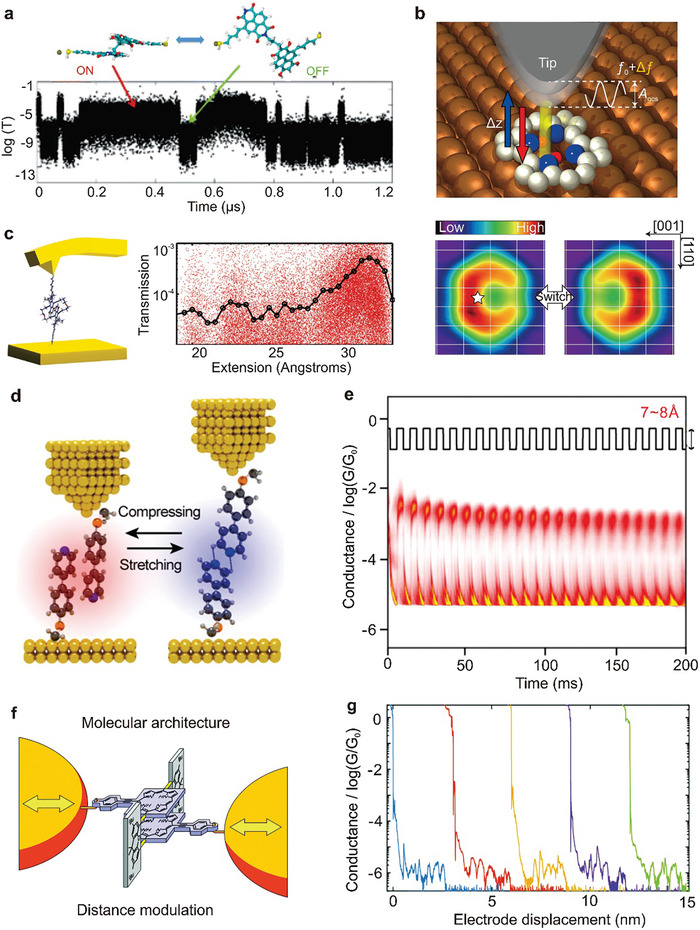
Several single molecule switches for molecular conformation change. a) As the molecule unfolds/refolds, the average conductance reversibly changes over three orders of magnitude. Reprinted with permission from ref. [222] Copyright 2011, American Chemical Society. b) STM images of a single porphycene molecule at 5 K (the white star indicates the lateral tip position during force spectroscopy) and the molecular structure of cis porphycene, which can be reversibly switched between the two mirrors reflected configurations (curved arrows depict hydrogen transfer during tautomerization). Reprinted with permission from ref. [223] Copyright 2016, Springer Nature. c) Schematic of a single‐molecule pulling/molecular electronics setup and the change of conductance corresponding to the molecular conformation change. Reprinted with permission from ref. [221] Copyright 2011, American Chemical Society. d) Illustrations of the PY‐2 supramolecular junctions formed by [π···π] and hydrogen‐bonding interactions. e) A 2D conductance histogram was constructed by aggregating conductivity‐time switching curves of PY‐2 at an oscillation frequency of 110 Hz. The black solid line represents the voltage amplitude applied on ceramic, corresponding to a distance change of 7–8 Å between two electrodes. At 110 Hz, the estimated conductance on/off ratio is ≈316. Reprinted with permission from ref. [224] Copyright 2023, American Chemical Society. f) A schematic representation illustrates the molecular structure positioned between two gold electrodes for distance modulation experiments. The depiction of the expanded porphyrin deck with acetylene‐based phenylthiol is presented in light blue, the flat bridging xanthine unit is depicted in gray, and the rotating acetylene joint is represented in light yellow. The attachment of the S‐Au bond and affixing the molecule onto the electrode is elucidated as a hook. g) Five examples of individual breaking traces, each displaced by hand by 3 nm to ensure good visibility. e,f) Reproduced with permission from ref. [225] Copyright 2021, The Royal Society of Chemistry.

Researchers have leveraged molecular dynamics simulations to manipulate molecules mechanically using a conductive atomic force microscope.^[^ ^222^ ^]^ The process involves transitioning the molecules from a folded to an extended state while monitoring their conductivity. As the molecules undergo folding and unfolding, their average conductivity exhibits a reversible three‐orders of magnitude change. However, the transport rate displays significant variation throughout the simulation, highlighting the necessity for statistical sampling in these systems. In the dynamic bistable region, it is anticipated that a novel single‐molecule feature with conductive flickering correlated with force flickering can still be observed. To better understand the system, the researchers established structure‐function relationships by plotting the dominant interactions among the molecules that mediated charge transfer during the stretching simulation process. **Figure** **6**a presents the conductance values of the molecule in both folded (ON state) and unfolded (OFF state) configurations, representing the switching state of the device. The folded state structure comprises two complementary aromatic rings – a naphthalene diimide and a pyrene tetrol – that strongly π‐stack due to the formation of four hydrogen bonds between the hydroxy (‐OH) and carbonyl (‐C_d_O) groups in the complementary aromatic units. The stacker is attached to the gold surface and the CAFM tip through thiol‐terminated alkene‐conjugated chains. In the unfolded conformation of the molecule, theoretical calculations predict a higher charge transfer barrier, resulting in a substantially lower conductivity.

Researchers have also discovered that, at zero bias and 5 K, simply bringing the probe near the molecule is enough to trigger *cis*↔*trans* isomerization.^[^ ^223^ ^]^ Figure 6b illustrates the experimental setup, where a tungsten tip mounted on a tuning fork sensor oscillates at its resonance frequency *f*_0_. The frequency shift Δ*f* is recorded as the tip approaches and retracts from a molecule (Δ*z*). The tip apex is expected to be covered by copper atoms, as it had been gently crashed into the bare surface several times before the measurement. The scanning tunneling microscope image of the molecule on a copper substrate reveals the conformational isomers of the molecule, as shown in the bottom panel of Figure 6b.

Theoretical reports reveal conformational changes experienced by molecules when subjected to mechanical force.^[^ ^221^ ^]^ These studies have introduced a unique model molecular system that exhibits non‐intuitive transport elongation behavior, which challenges our conventional understanding of molecular responses to force. As demonstrated in Figure 6c, an increase in the molecular stretching force leads to a corresponding rise in the tunneling current. The model molecule is composed of two complementary aromatic groups (1,4‐naphthoquinone and 1,4‐naphthoquinone), connected by two ether chains and anchored to a gold electrode through a thiolated terminal alkene.

To investigate the transport properties of this stretched molecule, researchers employed a combination of equilibrium molecular dynamics simulations under single‐molecule pulling and transport characteristics calculated by non equilibrium Green's function technique based on density functional at the Landauer limit. Contrary to the expected exponential decay of tunneling current with increasing molecular length, the simulation revealed a 10‐fold increase in electronic transport along the molecule as it stretched. This unexpected transport trend was elucidated through local current analysis, which unveiled the dual role of hydrogen bonding in both stabilizing π stacking at specific lengths and introducing additional electronic coupling between complementary aromatic rings. The simulation highlights a reverse mechanoelectrical single‐molecule switch based on a novel category of transport elongation behavior that can be achieved through mechanical manipulation. Furthermore, it underscores the significant sensitivity of conductance measurements to molecular conformation.

The latest research presents a strategy for constructing electronically robust switches by leveraging two distinct non‐covalent interactions between two pyridine derivatives.^[^ ^224^ ^]^ As depicted in Figure 6d, the formation of a compression junction results in the opening of a single supramolecular switch, wherein the π‐π interaction dominates transport. Conversely, closing the switch involves stretching the junction to form a hydrogen bond dimer, leading to a marked decrease in conductivity. This conductance decrease is attributed to the face‐to‐face stacking of the main chains of benzopyridine during compression bonding. Simultaneously, hydrogen bonding interactions arise from the formation of double [C‐H ··· N] hydrogen bonds between adjacent pyridine units when the connection is stretched.

To showcase the robustness of the supramolecular switch, periodic square waves were applied to the piezoelectric station, thereby modulating the tip‐substrate spacing. The movement distance of the tip correlates with the voltage applied to the piezoelectric ceramic. Consequently, various voltages were applied to determine the ideal electrode spacing for obtaining a suitable junction, as illustrated in the right panel of Figure 6e. This amplitude enables the complete extension of the supramolecular structure, promoting its preferred hydrogen bonding conformation within the nanogap. The graph indicates excellent reliability and reproducibility at an oscillation frequency of 110 Hz. Achieving robustness and reproducibility in individual supramolecular switches involved modulating the junction with Ångström precision at frequencies up to 190 Hz, yielding a high ON/OFF ratio of approximately 600. This study paves the way for designing robust bistable mechanical response devices, and poised for application in the construction of integrated circuits within microelectromechanical systems.

The mechanical stretching‐induced structural changes can induce quantum interference effects, thereby manifesting the characteristics of switching devices.^[^ ^225^, ^226^ ^]^ As depicted in Figure 6f, the intramolecular degree of π‐orbital overlap between porphyrins and the angle of the xanthine bridge relative to the porphyrin plane is manipulated to control electrical transport mechanically.^[^ ^225^ ^]^ Theoretical predictions of conductivity during the stretching process reveal an order of magnitude change, attributed to two potent destructive quantum interference features spanning the entire electron gap between the HOMO and the LUMO. In Figure 6g, the MCBJ experiment at room temperature corroborates the molecular junction's mechanical sensitivity response. Throughout the continuous stretching of molecules, noticeable conductivity changes of up to 1.5 orders of magnitude are observed during the breaking events, showcasing the most substantial reported mechanosensitive within cyclophane‐like systems. This study underscores the significant mechanical sensitivity of PC1 at room temperature, suggesting that in future investigations, two bridges could be connected on each side of the π‐stacked dimer to mitigate molecular deck distortion. Further exploration of molecular design is warranted to gain deeper insights into diverse mechanical‐sensitive responses and to create novel quantum sensors based on mechanically sensitive molecular systems.

### 5.2 Contact Geometry Change‐Induced Switches

Force can lead to a variety of structural modifications within chemically inert molecules or at the electrode‐molecule interface, enabling switching behavior.^[^ ^227^, ^228^, ^229^ ^]^ For example, a reversible binary switching device has been developed that can toggle between low and high conductance states by mechanically manipulating the Au‐4,4'‐bipyridine molecule‐Au junction.^[^ ^224^ ^]^ The variation in conductance is primarily attributed to different contact geometries at the flexible yet stable N‐Au bond, which directly affects the molecular junction's conductive properties.^[^ ^224^, ^230^ ^]^ Furthermore, recent studies employing the STM‐BJ technique have explored mechanical force‐induced switches incorporating thiophene groups.^[^ ^231^ ^]^ The observed changes in conductivity up to two orders of magnitude when the junction is compressed or stretched can be attributed to localized interactions between the weakly coordinating thiophene and the electrode. This facilitates reversible monodentate‐bidentate contact switching while allowing for the adjustment of the junction size. These findings not only contribute to our understanding of the conductance shifts in thiophene molecular junctions but also provide valuable insights into the development of force‐induced molecular switch devices.

**Figure 7:**
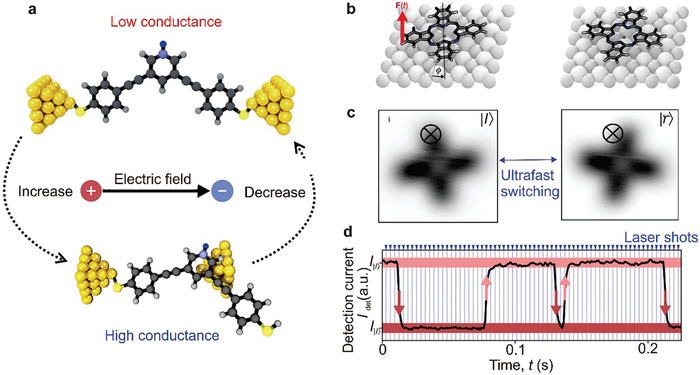
Single‐molecule switches with contact change. a) Schematics of electric‐field‐induced connectivity switching between meta‐and para‐connectivity. The para connectivity is expected to be favorable when large EEF is applied, owing to the counteracting of dipole moments with EEF. Reprinted with permission from ref. [144] Copyright 2020, Cell. b) In equilibrium, MgPc molecules are adsorbed on the NaCl lattice; applying an atomically local force stimulus F(t) might efficiently and coherently drive an in‐plane rotational oscillation of the molecule. c) Low‐voltage maps of the tunneling current at constant height display the in‐plane rotation of the neutral molecule in both adsorption geometries. d) During repetitive terahertz pulse irradiation (blue vertical lines) of the STM tip, the orientation of the molecule can be monitored using non‐resonant detection current *I*_det_ (black), which statistically triggers back‐and‐forth switching between the adsorption geometries (arrows). b–d) Reproduced with permission from ref. [232] Copyright 2020, Springer Nature.

Both mechanical force and electric field force can alter the interaction state at the interface between the electrode and the molecule. By manipulating the molecular dipole and electric field force, it is possible to modify the contact points between molecules and electrodes, ultimately affecting the conductivity of molecular devices.^[^ ^227^ ^]^ Hong et al., made a remarkable discovery that the pyridinium ring can interact with gold electrodes, leading to the development of a molecular framework with pyridine at its center. This design enabled two distinct connectivity: end‐to‐end meta‐connectivity and in‐backbone para‐connectivity, which correspond to low and high conductivity states respectively, as illustrated in **Figure** **7**a. This connectivity is controlled by protonation and the applied bias between the electrodes, highlighting the critical role of dipole moment interactions and electric fields in modulating the connectivity of single‐molecule junctions. Furthermore, switching between meta‐ and para‐connectivity resulted in a change in electron transport distance, transitioning from longer to shorter pathways. This increased the conductivity difference between the two connectivity, enabling the reversible switching of linkage within the same molecular backbone. This innovative approach offers a new paradigm for the efficient manipulation of single‐molecule junctions.

Additionally, the application of a femtosecond pulse to the atomic‐level STM tip allows for the mechanical rotation of molecules adsorbed on a substrate.^[^ ^232^ ^]^ Concurrently, electrons are injected into the LUMO energy level of the molecule at various angles. This injection causes the change in conductance at different rotation angles, ultimately realizing molecular switch devices shown in Figure 7b. Researchers have investigated the switching behavior of a molecule relative to a sodium chloride substrate by analyzing its response to a ±10° rotation around its axis. This rotation corresponds to a neutral state with no switching characteristics. To study the switching probability, the researchers employed ultrafast, time‐delayed electron injection events using STM driven by femtosecond light waves. They focused the carrier of the laser pulse onto the STM junction, setting an instantaneous bias voltage that created a femtosecond time window of ≈100 fs. Upon electron injection, an instantaneous rotation of the molecule toward 0° was triggered, leading to a different state as depicted in Figure 7b. The activation of the molecular switch only occurred through resonant tunneling into or out of one of the molecular orbitals, causing temporary charging.

As illustrated in Figure 7c, the single‐molecule switch toggled with a certain probability by femtosecond electron injection through a single‐shot operation of a terahertz laser source. The adsorption geometries |*l*⟩ and |*r*⟩ were monitored in steady‐state imaging at low bias voltages without switching between them. By positioning the STM tip above the molecule so that the tunneling current varied for |*l*⟩ and |*r*⟩, researchers could use the detection current *I*_det_ to continuously monitor the adsorption geometry without taking images. Figure 7d shows a typical time trace of *I*_det_ acquired over 0.23 s while single terahertz pulses repeatedly injected electrons into the LUMO. Whenever an electron injection triggered the molecule to switch its adsorption geometry, the current sharply changed from I|*l*⟩ to I|*r*⟩ or vice versa, allowing for high‐accuracy detection of every switching event. By registering all events separately for both directions (as indicated by the arrows in Figure 7d), researchers could differentiate the probability of switching from *l*⟩ to *r*⟩ and the reverse probability p_r‐l_ for each light pulse.

### 5.3 Charge State Change Induced by Force

The alteration in the charge state involves variations in the molecular form under external stimuli, such as the spin state and the redox state of the molecule. Traditional methods to induce changes in molecular states primarily involve the MCBJ or STM techniques.^[^ ^233^ ^]^ The transport of a mechanically triggered single‐molecule switch based on the spin state dependence of Fe^II^ ion‐coordinated spheres was investigated using MCBJ technology.^[^ ^234^ ^]^ In these molecules, the relative arrangement of two trispyridine ligands within the same Fe^II^ complex can be mechanically controlled under specific junction configurations. Mechanical stretching has the potential to distort the Fe^II^ coordination sphere, ultimately altering its spin state. To support the hypothesis‐based switch mechanism involving spin‐crossover behavior, low‐temperature current‐voltage measurements were performed at the mechanically controlled breaking point using movable nanoelectrodes. Remarkably, most of the molecular junctions formed with spin‐crossover‐active Fe^II^ complexes exhibited increased conductivity corresponding to the widening electrode distance, reaching up to 1–2 orders of magnitude. Theoretical calculations further predicted a higher transmission rate for stretch‐induced spin transitions and high‐spin configurations in Fe^II^ complexes.

A core concept of the electron transfer theory is the coupling between the electronic state of a molecule and its structure.^[^ ^22^ ^]^ Experimental evidence demonstrates that mechanical stretching modifies the metal‐ligand bond length within a single metal complex molecule and subtle variations in molecular structure can lead to alterations in electronic energy levels. These changes effectively guide electron transfer reactions in a predictable manner, resulting in shifts in oxidation states and the manipulation of single‐molecule conductivity.

**Figure 8:**
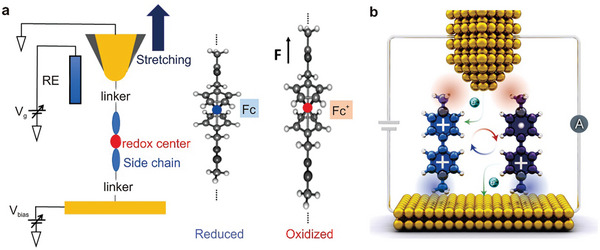
Molecular switches with charge state change. a) The experimental setup depicted involves a redox molecule bridging between a gold STM tip and a gold substrate, which serves both to interrogate the redox state of the molecule via conductance measurement and to stretch the molecule. Reproduced with permission from ref. [22] Copyright 2017, American Chemical Society. b) The STM‐BJ method demonstrates that the pyridinium groups efficiently serve as electrostatic anchors, leading to the formation of robust gold‐molecule‐gold junctions. The method shows the electron injection‐induced redox switching that occurs in single‐molecule junctions. Reproduced with permission from ref. [233] Copyright 2021, American Chemical Society.

**Figure** **8**a illustrates the use of a gold needle and a gold substrate as two electrodes, with the molecule's geometric shape being altered by mechanical stretching while its conductivity is measured. To test or refute the hypothesis that stretching is linked to changes in oxidation‐reduction states, researchers conducted a series of experiments. They found that stretching had no discernible effects on the conductivity changes associated with electronic coupling. The observed increase in conductivity due to stretching was primarily evident in molecules near the oxidation‐reduction potential. The low and high conductivity levels corresponded to the reduced and oxidized states, confirming that the conductivity conversion is related to the change in oxidation‐reduction state rather than molecular conformation.

This observation supports the notion that mechanical stretching alters bond lengths and promotes reactions toward the final state favorable for bond length changes, inducing oxidation‐reduction reactions in single metal complex molecules. This finding implies that mechanical stretching can control the oxidation‐reduction states of individual molecules, offering a method for mechanically manipulating their electronic properties. This study promotes the development of molecular machines with electromechanical functions, providing a solid foundation for understanding molecular switch devices driven by force‐induced charge state changes.

The charge transport in single‐molecule junctions depends on the chemical identity of the anchor groups used to connect the molecular wires to the electrodes. A new anchoring strategy has been reported through the efficient Coulombic interaction between gold electrodes and positively charged pyridinium terminal groups.^[^ ^233^ ^]^ Furthermore, it examines electron injection‐induced redox switching in single‐molecule junctions under the influence of an electric field. This anchoring approach and redox‐switching mechanism have the potential for a new class of redox‐activated single‐molecule switches.

Figure 8b shows a study using electrostatic interactions to create stable molecular connections between Au electrodes and positively charged methyl pyridine terminal molecules.^[^ ^233^ ^]^ Using custom‐made STM‐BJ technology, researchers compared the conductivity and charge transfer behavior of neutral and charged pyridine terminal single molecule connections. The dimethylbiszine molecule had a binary conductive state, consisting of the ground state with two cations and the metastable state with single electron radical ions, which was attributed to a redox reaction triggered by electron injection/extraction in the structure. To investigate the redox switching mechanism, researchers used spectroscopic electrochemical experiments and electrochemical surface‐enhanced Raman spectroscopy (EC‐SERS) to elucidate the role of the electric field in electronic catalysis.

Additionally, the AC/DC module of COMSOL Multiphysics was used to simulate the electric field distribution in the fracture structure and assess the local electric field focusing on STM‐BJs. The simulation results showed that electron injection and reduction occur only when molecules are trapped in structures with small gap sizes and high electric field strength, while direct tunneling occurs through the molecule's ground state otherwise. This anchoring strategy and redox switching mechanism may serve as the foundation for a novel type of single‐molecule switch.

## 6 Other Molecular Switches

To provide devices with switching capabilities, several additional incentives can be used in addition to the previously mentioned stimuli for achieving switching characteristics. These include spin switches, stereoelectronic switches, and chemically stimulated switches. In the following sections, we will mainly address molecular switches that utilize these three stimulation methods as representative examples.

### 6.1 Spin‐Induced Switches

The molecular spin switch is an intriguing approach for controlling spin polarization at the interface between molecules and magnetic metal electrodes in molecular spintronic devices. This emerging field combines spintronics and molecular electronics.^[^ ^235^, ^236^ ^]^ By manipulating both spin and charges, molecular spintronic devices containing one or more magnetic molecules exhibit potential applications in information storage and processing.^[^ ^237^ ^]^ Notably, single‐molecule magnets (SMMs) have been explored for their rich quantum behaviors,^[^ ^238^ ^]^ such as quantum tunneling of magnetization, quantum phase interference, the Kondo effect,^[^ ^239^, ^240^ ^]^ and the Zeeman splitting effect.^[^ ^241^ ^]^ Specifically, for double‐decker phthalocyanine (Pc) molecules with SMM properties, the spin in a delocalized molecular orbital can interact with metal electrode conduction electrons, forming a unique Kondo resonance that significantly differs from the Kondo resonance created by metal spins.^[^ ^242^ ^]^ SMMs with large spins and high anisotropy barriers exhibit magnetic hysteresis effects, presenting an exciting opportunity to utilize spins for information storage and processing.

The magnetic or spintronic properties of switchable molecular spin systems can be controlled, offering potential applications in fields such as data storage and spintronics. For instance, an electric current applied through an STM can switch the molecular spin on and off, causing the disappearance and reappearance of the Kondo resonance resulting from the coupling between a localized electron spin on the molecule and conduction electrons in the metal electrodes.^[^ ^243^, ^244^ ^]^ Moreover, the surface magnetism of adjacent substrates can also influence the performance of SMMs. The spin‐orbit coupling of molecules with spin characteristics can be manipulated by an electric field to control the electron spin, enabling tailored design through suitable fabrication methods. Furthermore, the spin direction of such molecules can be adjusted by applying stress or an external electric field, leading to either high or low conductance states and thus achieving a switching effect. Notable examples of such metal complexes include phthalocyanine and azopyridine molecules.^[^ ^243^, ^245^ ^]^

This study presents the induction of spin‐state switching in individual Ni‐porphyrin molecules in a homogeneous solution at room temperature using light‐driven coordination‐induced spin state switching (LD‐CISSS).^[^ ^245^ ^]^ The switching mechanism involves converting the Ni‐porphyrin ligand between high and low spin states via ultraviolet (365 nm) and visible light (455 nm) stimulation. The square planar platform of nickel porphyrin is utilized, with azopyridine serving as an axial ligand for photoisomerization. Since the square planar nickel porphyrin is diamagnetic (low spin state, S = 0), all complexes with axial ligands are paramagnetic (high spin state, S = 1). Consequently, irradiation with UV light (*trans*‐*cis*) and visible light (cis‐trans) can induce spin state switching, rendering the structure a functional spin switch. This research expands the potential application areas for spin‐magnetic materials, such as modulating the performance of isolated molecules on surfaces or the relaxation of contrast agents employed in magnetic resonance imaging.

Recently, the same group developed the molecular spin switches using the Ni‐porphyrin as in previous studies.^[^ ^246^ ^]^ In this case, however, they integrated the spin‐switching ligand into a more robust complex by utilizing the mechanical movement of the axial ligand bound to the porphyrin ring. The stability of the two spin and coordination states of this molecule extends beyond a few days at low temperatures (4 K). The potential applications of this switch concept extend beyond spin functionality, with possible specific applications in controlling the catalytic activity of surfaces in the future.^[^ ^246^ ^]^

**Figure 9:**
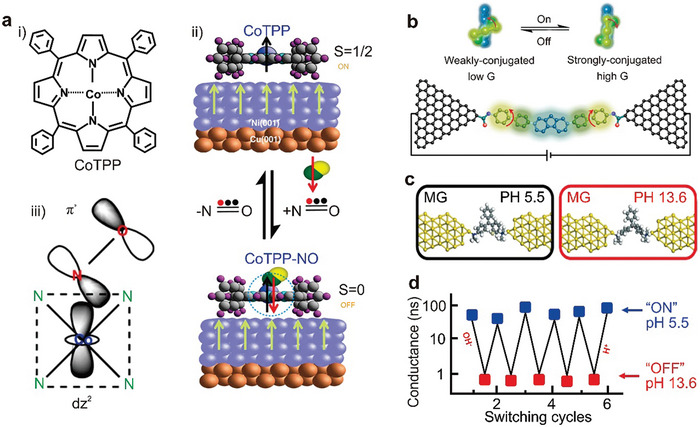
Several other types of single molecule switches. a i) Chemical structure of the CoTPP molecule. ii) The switching process of CoTPP molecules between ON and OFF states: ON state (top: S = ½); OFF state (bottom: S = 0). iii) A NO – CoTPP complex scheme depicting the relevant orbitals' involvement. Reproduced with permission from ref. [247] Copyright 2010, Springer Nature. b) Schematic conformational transition of terphenyl units between strongly‐conjugated and weakly‐conjugated states and corresponding hexaphenyl aromatic chain single‐molecule junctions (The red arrows represent the direction of rotation of the molecule). Reproduced with permission from ref. [251] Copyright 2017, American Chemical Society. c) The two probe structures are used for the electron transport calculations of MG molecules under two different pH values. d) Conductivity modulation of MG molecules between “ON” and “OFF” states, which is caused by different pH values. c, d) Reproduced with permission from ref. [259] Copyright 2014, Wiley‐VCH GmbH.

The development of chemical systems featuring switchable molecular spins holds the potential to create materials with controllable magnetic or spintronic properties. Studies have shown that an organometallic molecule coupled to a ferromagnetic substrate can be toggled between magnetic off and on states by a chemical stimulus.^[^ ^247^ ^]^ **Figure** **9**a(i) shows the use of a magnetic molecule, cobalt‐porphyrin (CoTPP), characterized by one unpaired electron (S = ½), as a candidate for a magnetic system influenced by the up or down magnetization of a ferromagnetic nickel substrate. The architecture of the CoTPP molecule consists of a porphyrin complex and a magnetic cobalt atom.

Figure 9a(ii) depicts the mechanism of nitric oxide (NO)‐induced reversible switching of the magnetic response of CoTPP coupled to a ferromagnetic Ni substrate. The CoTPP spin is magnetically coupled to the ferromagnetic substrate (nickel (Ni (001))) and can be modulated by NO, which serves as a chemical stimulus. The introduction of NO supersedes the Co‐Ni magnetoelectronic interaction by forming a NO‐CoTPP nitrosyl complex, corresponding to the off state of the Co spin in CoTPP. Thermal dissociation of NO from the nitrosyl complex reinstates the initial Co‐Ni magnetoelectronic interaction and results in the on the state of the Co spin in CoTPP. Consequently, the mechanism aligns with NO reacting with CoTPP from the top and forming the NO‐CoTPP complex. This research offers an example of a chemical switch affecting the spin in surface‐adsorbed molecules. It presents a case for the spin *trans* effect in analogy to the well‐established *trans* effect.

Density functional theory calculations suggest that magnetic exchange coupling requires the metalloporphyrin molecule to assume a planar orientation, as shown in Figure 9a(iii), with the metal ion‐4N moiety (represented as ‘Co‐4N’ in Figure 9) on the surface and maintained at a predetermined distance from the magnetic substrate. The NO‐induced reversible switching of surface‐adsorbed molecular spins is attributed to the spin *trans* effect, which governs the spin localized at a central metal ion. In addition to the spin *trans* effect, spin crossover phenomena dependent on electrostatic fields represent another mechanism for achieving spin switching. The novel spin switch relies on the E‐field‐dependent coordination sphere of Fe^II^ terpyridine complexes with MCBJ in a vacuum at liquid helium temperature.^[^ ^248^ ^]^ Researchers fabricated the molecular junction with Fe^II^ (tpy)_2_ complexes, and the switching behavior was observed due to the ligands' spatial arrangement caused by the sensitivity of the molecules' spin states.

### 6.2 Stereoelectronic Switches

The stereoelectronic effect refers to the phenomenon in which a molecule's structure influences the distribution and configuration of its electron cloud, thereby affecting the process and rate of chemical reactions. This effect arises from differences in molecular structure, where the spatial relationships between various groups and atoms within the molecule impact the distribution and configuration of the electron cloud. These internal structural effects can alter the molecule's reactivity and reaction mechanism, ultimately influencing the rate and selectivity of the reaction. Consequently, understanding the nature of the stereoelectronic effect can guide molecular conformations, enabling devices to achieve switching performance.^[^ ^249^, ^250^ ^]^

Stereoisomerization is a phenomenon observed in the *tran*s‐*cis* isomerization of azobenzene molecules and fully methylated oligosilanes with a methylthio electrode junction. The stereoelectronic properties of the sulfur‐methylene σ bond of the terminal molecule are significantly influenced by the strong σ‐conjugation in the oligosilane backbone. Theoretical calculations support the existence of three distinct dihedral conformations, each possessing markedly different electronic properties. By simply stretching or compressing the molecular linkage, researchers can switch between these three conformations, enabling the digital switching of molecular junction conductivity between two states.^[^ ^18^ ^]^

Researchers have developed a method to study the structure of graphene‐molecule single‐molecule junctions using a specially designed hexaphenyl aromatic chain molecule to create a stable single‐molecule structure by covalently sandwiching it between nano‐gapped graphene point contacts, as depicted in Figure 9b.^[^ ^251^ ^]^ This specific hexaphenyl aromatic chain molecule allowed the observation of varying degrees of conjugation resulting from the thermally induced twisting of the phenyl rings in the aromatic chain. In the terphenyl molecule, two distinct dihedral angles were identified between the outer benzene rings at each end. One angle is approximately zero, while the other is nearly double that observed between the benzene rings in biphenyl. It is hypothesized that the greater overlap of the π‐π orbitals in the former configuration results in stronger conjugation, referred to as the “strongly conjugated” state, while the latter state, with weaker overlap and conjugation, is termed the “weakly conjugated,” as shown in the upper panel of Figure 9b. The researchers also observed a temperature dependence in the twisting of the phenyl rings, as evidenced by the *I‐V* characteristics measured over a temperature range from 100 to 140 K. Specifically, increment in the temperature led to a higher frequency of conductance switching, while the ratio of the current between the high and low conductance states gradually decreased.

Overall, this study provided a reliable approach to explore the stereoelectronic effect on molecular conductivity in a specially designed hexaphenyl aromatic chain molecule. By covalently linking the aromatic chain molecule to graphene electrodes, forming robust single‐molecule junctions, a temperature‐dependent stochastic conductance switching mechanism was demonstrated, originating from the thermally‐induced random twisting of the phenyl group. This mechanism resulted in the strongly conjugated (high conductance) and weakly conjugated (low conductance) states verified experimentally and theoretically. These findings offer a new perspective on designing practical single‐molecule devices based on stereoelectronic effect‐induced conductance switching and suggest potential applications in developing high‐performance organic functional materials for electronic and optoelectronic applications, particularly those based on biphenyl.

### 6.3 Chemical Control Switches

Chemical stimulants, such as acids, bases, metal ions, and chemical reactants, have the capacity to alter the conductance of molecules.^[^ ^195^, ^212^, ^252^, ^253^, ^254^ ^]^ This alteration can occur through adjustments in molecular conformation or by initiating chemical reactions. The molecular conformation changes induced by these stimulants can result in alterations to the molecule's electronic structure, subsequently modifying its conductance properties. Similarly, chemical reactions initiated by these stimulants can cause changes in molecular structure, ultimately impacting its electrical properties. Overall, these chemical stimulants hold the potential for significant contributions to the development of new molecular electronics and related fields.^[^ ^255^, ^256^ ^]^

Examples of chemically stimulated molecular switches include azobenzene molecules, dye molecules, and malachite green (MG) molecules, which undergo conformational changes to achieve switching characteristics. A common chemically stimulated molecular switch involves changing the pH value of a solution to alter the channel's conformation and achieve switch characteristics.^[^ ^119^, ^257^ ^]^ For instance, molecules containing sulfonic acid groups have been employed to achieve a reversible switching effect, allowing distinct conductance states to be initiated through exposure to different pH values. The azobenzene molecule, which contains two sulfonic acid groups, can react reversibly with both base and acid, making it sensitive to various pH values.^[^ ^258^ ^]^ This finding suggests the potential for integrating multiple functionalities within a single molecular device.

In contrast to the previously mentioned study, a novel single‐molecule switch capable of detecting pH has been developed. This switch is based on the pH‐induced conjugation and electronic alterations of dye molecules, which can be observed through STM.^[^ ^259^ ^]^ There are reports exploring the concept of a single‐molecule pH electric sensor through the deliberate selection of MG and para‐phenylenediamine (PA) dyes as molecular sensing units. As pH indicators, these two molecules exhibit significant color differences when the pH value of the solution changes, suggesting that electronic perturbations are often induced by pH‐induced alterations in molecular structure. In this study, MG is protonated and in a conjugated form in neutral or slightly acidic solutions. However, in alkaline solutions (pH > 13), MG loses its internal proton and adopts a non‐conjugated structure, as illustrated in Figure 9c.^[^ ^259^ ^]^ Due to the reversible structural transformation between conjugated and non‐conjugated states, pH‐induced electronic modulation occurs, resulting in a change in the HOMO‐LUMO gap. Consequently, the m‐M‐m device mediated by MG molecules becomes highly responsive to the environmental pH, exhibiting a high switching ratio (≈100:1) in device conductivity, as shown in Figure 9d.

### 6.4 Neuromorphic Switches

Neuromorphic switches refer to a class of electronic devices that mimic the functionality of biological neurons and synapses.^[^ ^260^ ^]^ These devices are designed to perform cognitive tasks such as learning, recognition, and decision‐making, similar to how the human brain operates.^[^ ^261^, ^262^, ^263^, ^264^ ^]^ While these may not be examples of single‐molecule devices, they represent examples of molecular‐scale switching with dynamic properties, which is an interesting new direction. The field of neuromorphic computing aims to develop hardware that can process information in a brain‐like manner, offering advantages in terms of speed, energy efficiency, and adaptability compared to traditional computing architectures. Research on neuromorphic switches includes the development of novel materials and devices that can emulate the synaptic and neuronal functions of the brain.

One key aspect of this research is the design of memristive devices, which exhibit resistance switching behavior analogous to the synaptic plasticity observed in biological synapses. Memristors can change their resistance in response to applied voltage pulses, allowing them to store and process information in a manner similar to the way synapses in the brain change their strength in response to electrical signals. Yu et al. reported a universal dynamic mode of molecular devices that solves the transient redox state of quinone molecules commonly present in junctions through proton/water transfer.^[^ ^265^ ^]^ Diffusion constraints on slow proton/water transfer modulate fast electron transfer, leading to non‐stationary transport processes characterized by negative differential resistance, dynamic hysteresis, and memory‐like behavior. By integrating theoretical models with transient characteristics, a quantitative paradigm for studying the dynamics of non‐stationary charge transport has been further developed. Additionally, the principles of dynamic devices have been elucidated through numerical simulations. Ahead of this report, Nijhuis et al. reported a molecule exhibiting a significant negative memory resistance behavior, transitioning from a high conductivity state to a low conductivity state, depending on the driving speed and the number of past switching events.^[^ ^266^ ^]^ This dynamic molecular switch emulates synaptic behavior and Pavlovian learning, all within a 2.4 nm thick layer, which is three orders of magnitude thinner than neuronal synapses. Dynamic molecular switches offer all the fundamental logic gates necessary for deep learning due to their temporal and voltage‐dependent plasticity. The multifunctional dynamic molecular switch, mimicking synapses, represents an adaptive molecular‐level hardware that can be integrated into solid‐state devices, paving the way for simplifying dynamic and complex electrical operations encoded within a single, ultra‐compact component.

Another research area of neuromorphic switches involves the development of spiking neural networks (SNNs), which are computational models inspired by the way neurons communicate in the brain. SNNs use spikes, or brief pulses of activity, to encode information, and they can perform tasks such as pattern recognition and decision‐making with high efficiency. Overall, research on neuromorphic switches aims to create brain‐inspired computing systems that can offer significant improvements in terms of efficiency, speed, and adaptability compared to conventional computing paradigms. Saptarshi et al. demonstrated a biomimetic device based on a dual‐gate MoS_2_ field‐effect transistor (FET) capable of encoding analog signals into random spike sequences using various neural encoding algorithms.^[^ ^267^ ^]^ These algorithms include rate‐based encoding, spike timing‐based encoding, and spike counting‐based encoding. The researchers successfully captured two important aspects of neural coding: dynamic range and coding accuracy. Moreover, they found that the encoding energy was saved to ≈1–5 pJ per spike. Furthermore, the researchers demonstrated the fast encoding of the MNIST dataset using biomimetic devices, achieving over 91% accurate inference using trained spiking neural networks (SNNs) in ≈200 timesteps.

## 7 Metal Atom Switches

Atomic switches are nanoscale devices that allow for the control and manipulation of single atoms using external stimuli such as electric fields, light, or electrochemistry. The term of the “atomic switch” was first introduced by Eigler et al.^[^ ^268^ ^]^ They demonstrated resistive switching in one order of magnitude by controlling an atom transfer between the tip of a scanning tunneling microscope and a sample surface depending on the adsorption site of the transferred atom. As part of the nanotechnology domain, these switches have the potential to revolutionize the manufacturing of ultra‐small circuits, such as microprocessors in computer chips. The key advantages of atomic switches include their minuscule size, low power consumption, and high reliability and precision due to their single‐atom composition.

Atomic switches are being developed using various approaches, including mechanical tuning,^[^ ^269^, ^270^ ^]^ bias voltage tuning,^[^ ^271^, ^272^ ^]^ and electrochemical methods.^[^ ^271^, ^273^ ^]^ The phenomenon of quantum conductivity, resulting from reducing a metal wire's cross‐section to a few atoms,^[^ ^274^ ^]^ demonstrates the potential of active nanodevices like atomic switches. However, definitive proof of switching caused by the movement of a single atom or a few atoms remains lacking. To achieve active nanodevices, control over metal junctions, semiconductor structures, or single atoms in electrochemistry is crucial.

Atomic switches can be categorized into two types based on atomic point contact. The first type of atomic switch relies on changes in the electrode gap or the rearrangement of atoms triggered by an electric or light field.^[^ ^274^, ^275^, ^276^, ^277^, ^278^ ^]^ These switches utilize the movement or reconfiguration of atoms in response to external stimuli to achieve switching capabilities. The second type of atomic switch involves the migration of ions to form a metal bridge within the device, enabling switching through electrochemical processes.^[^ ^279^, ^280^, ^281^, ^282^ ^]^ These switches depend on the controlled movement of ions to create or break connections between electrodes, thus regulating the flow of current.

### 7.1 Light‐Driven Atomic Switches

Light‐driven atomic switching is a process that involves the formation of direct atomic‐level contact conductance between electrodes through the thermal expansion of the electrode or substrate.^[^ ^99^, ^275^ ^]^ This expansion of electrodes is caused by surface plasmon polaritons (SPPs) at the nanogap, which are induced by light irradiation, resulting in the connection or open state of the switch.^[^ ^275^ ^]^ To achieve the off state, the light is turned off, allowing heat to dissipate, leading to the disconnection of the electrodes and a sharp reduction in conductivity.

**Figure 10:**
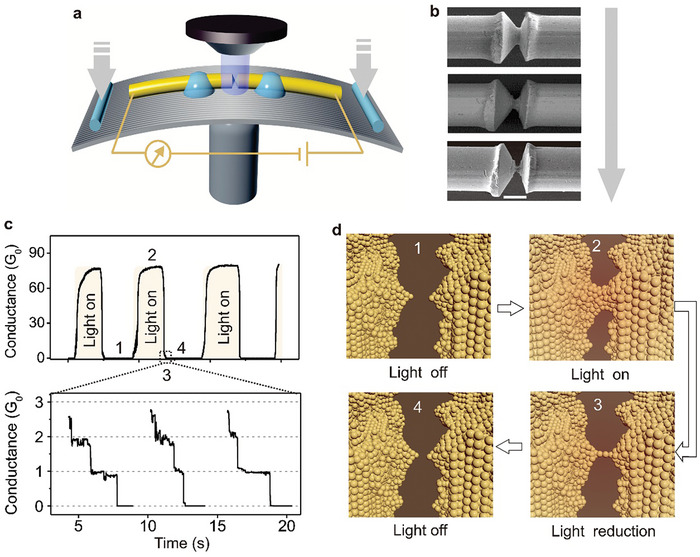
Strategy for the realization of atomic switching and conductance regulated by light illumination. a) Schematic diagram of the device structure. b) SEM images of the notched microwire during the stretching process. Scale bar: 50 µm. c) Real‐time current measurement of the junction upon light illumination with an interval of 50 s – 60 s. Zoomed image: conductance decreases in quantum steps at multiples of G_0_ (= 2e^2^/h) as the light intensity decreases. d) Schematic of the atomic arrangement corresponds to four conductance states upon light illumination. Reproduced with permission from ref. [275] Copyright 2019, Springer Nature.

Surface plasmons (SPs) are coherent delocalized electron oscillations that occur at the interface between two materials. They can be excited by concentrating light into subwavelength gaps between two metallic nanostructures. When the resonance frequency of SPs matches the frequency of the incident light, strong light absorption and significant plasmonic heating occur. The connection and disconnection of metal electrodes can be achieved through the thermal expansion of metal electrodes due to plasmonic heating.^[^ ^283^ ^]^ A local surface plasmon resonance (LSPR) is formed when a specific wavelength of light matches the metal electrode, creating a strong electric field that enhances heat generation and causes thermal expansion of the metal electrode. This expansion ultimately results in the formation of metal atomic contact. Turning off the light leads to the disconnection of the atomic connection, and this process can be repeated. As a result, precise regulation of the metal electrode gap at the atomic level can be achieved by adjusting the light intensity, enabling control over the conductivity of the metal point contact, which can range from 10^−6^ G_0_ to 10^2^ G_0_. **Figure** **10**a illustrates the strategy for forming a nanogap between metal electrodes based on the MCBJ technology (refer to Chapter 2 for details). Figure 10b displays a scanning electron microscope (SEM) image of a gold metal wire during the mechanical stretching process, demonstrating a progressive decrease in constriction cross‐section until the wire breaks, resulting in two separated electrodes.

At the beginning of the experiment, the gap between the two electrodes was set to a few angstroms (10^−5^ G_0_ < G < 10^−4^ G_0_) by the control of the MCBJ push rod. When the light was turned on, the conductance of the system increased from an initial value of G ∼ 10^−5^ G_0_ to a stable value of G ∼80 G_0_. Turning off the light caused the conductance to decrease to a value far below 1 G_0_ (close to the initial 10^−5^ G_0_). This conductance switching behavior was consistently observed in each measurement period, as shown in Figure 10c. Conductance decreases in quantum steps at multiples of G_0_ (= 2e^2^/ћ) was clearly observed as the light intensity decreased. Figure 10d presents the atomic arrangement under light illumination and light extinguishing. Initially, the two electrodes were separated by a few angstroms (state 1). Nanogaps exhibited strong light absorption due to localized plasmon resonances, and considerable thermal energy is generated. State 2 displays the reconnection of two electrodes due to electrode expansion driven by the thermal energy of LSP. Decreasing light intensity (cooling of the electrodes) led to metal wire shrinking and forming an atomic point contact before nanocontacts broke (state 3). Turning off the light separated the electrodes again, and electron transport returned to the tunneling regime (G ≪ 1 G_0_) (state 4).

**Figure 11:**
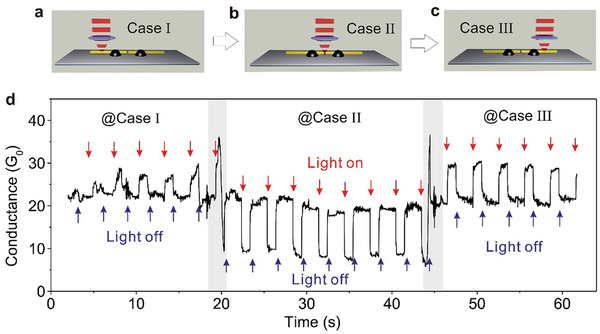
Real‐time conductance measurement of the gold wire when laser irradiates different spots of the wire. The light irradiates the a) left part of the gold microwire fixed by two drops of glue on the substrate, b) the central constriction of the gold wire, and c) the light irradiates the right part of the wire. d) The current response of the gold wire circuit when different parts of the wire are irradiated by the laser. The red arrows indicate the beginning of the laser irradiation, and the blue arrows indicate the turning off the laser. The two shaded gray regions indicate the transition regimes in which the irradiation spots are changed. Reproduced with permission from ref. [276] Copyright 2021, Elsevier Ltd.

Meanwhile, the same group further found that the pair of electrodes above the substrate can be driven to connect or disconnect even when the laser irradiates the substrate rather than the nanogap.^[^ ^276^ ^]^ Additionally, they demonstrated that the direction of the atomic conductance switch (*i.e*., conductance increases or decreases during light irradiation) can be accurately controlled by adjusting the irradiation position of the focused laser, as shown in **Figure** **11**.

The simulations demonstrated that the primary mechanism for this conductivity modulation is the thermal expansion of the substrate under the suspended electrodes which induces the relative displacement of the two electrodes. Briefly, when the light irradiates an area between the two glue drops (Figure 11b), the nanocontact will be stretched (current decreases) due to the opposite movement of the two glue drops driven by thermal expansion of the substrate. In contrast, when the laser irradiates an area outside the two glue drops (Figures 11a,c), the nanocontact will be compressed (current increases) due to the unidirectional but unequal movement of the two glue drops upon light irradiation, as shown in Figure 11d.

### 7.2 Electric Field Driven Atomic Switch

Electrically‐driven atomic switches represent a technique for controlling electron flow at a single‐atom or molecular scale by creating an electron transport channel through the application of electric fields or voltages. Atomic bridges can be formed via two primary methods: (1) migration and rearrangement of atoms driven by electric field and light irradiation,^[^ ^274^, ^277^, ^278^ ^]^ or (2) formation of a metal atomic bridge/filament through an electrochemical reaction.^[^ ^284^ ^]^ Electromigration is a well‐established technique for reducing the size of mesoscopic wires to the atomic scale or for generating nanometric gaps between pairs of metal electrodes. This process enables metal atoms to migrate under a strong electric field, allowing the rearrangement of metal atoms in the gap and forming an atomic electric switch capable of making or breaking atomic contact. Recent studies have reported the fabrication of atomic switches using electric field‐assisted technology.^[^ ^11^, ^274^, ^277^, ^278^, ^285^ ^]^

**Figure 12:**
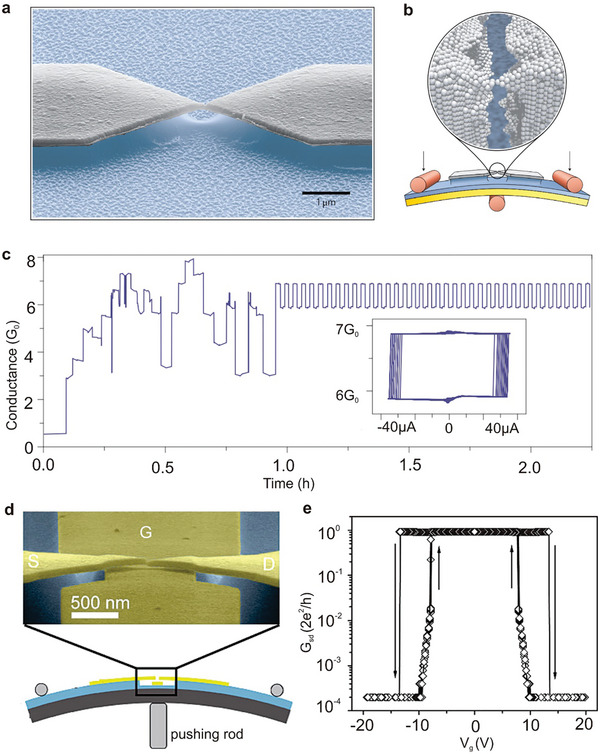
Strategies to realize atomic switches. a) Scanning electron microscope image of a break‐junction sample. b) Work principle of the break‐junction set‐up. c) Conductance of a break‐junction structure made from aluminum as a function of time when applying the control current given. Inset: conductance versus control current for the bistable part. a‐c) Reproduced with permission from ref. [274] Copyright 2013, Springer Nature. d) Schematic of the three‐point bending MCBJ setup with the bottom gating electrode. e) Switching characteristics of the break junction during 20 consecutive gating voltage cycles. d,e) Reproduced with permission from ref. [11] Copyright 2009, American Chemical Society.

Schirm et al. show that a metallic atomic‐scale contact can be operated as a reliable and fatigue‐resistant two‐terminal switch.^[^ ^274^ ^]^ A few‐atom aluminum contact is formed using a lithographically defined MCBJ at ultra‐low temperatures, as demonstrated in Figure 12a,b. To initialize the switch, the authors prepared contacts with a conductivity range of several G_0_ by bending the substrate. They then increased the bias current and monitored the conductance of the contacts simultaneously. Whenever a sudden change in conductivity (ΔG > 0.125 G_0_) occurs, the ramp direction of the current is reversed with a changing rate proportional to the step height. After 10–20 steps of irregular jumping height training, the probability of bistability reached 15%. Once the bistable state was achieved, it typically lasted for several hours and more than 500 switching cycles. **Figure** **12**c displays the conductance of a break‐junction as a function of time when applying the control current. Owing to its hysteretic behavior with two distinct conductance states, this two‐terminal switch can be used as a non‐volatile information storage element.

The MCBJ‐based method can also enable atomic‐level connections and disconnections between metal electrodes by applying a gate voltage with a three‐terminal apparatus.^[^ ^11^, ^286^ ^]^ Figure 12d shows a nano‐electromechanical relay, which exhibits high conductivity in its active state in a vacuum.^[^ ^287^ ^]^ Figure 12d presents a scanning electron micrograph of a gated MCBJ. Before the measurements, the devices were mounted in a three‐point‐bending setup and the sample chamber was evacuated to 10^−5^ bar before cooling in liquid helium at ≈6 K. The electrostatic attraction of the gate to the source and drain causes a deflection in the electrode tip and subsequently changes the electrode separation, which can be employed to destroy and form monatomic contacts. Figure 12e shows the switching characteristics during 20 consecutive gate voltage cycles. The source‐drain conductance of the junction reproducibly switches between the tunneling regime and a single‐atom contact with a conductance of approximately 1 G_0_.

Graphene's excellent mechanical and electrical properties combined with its compatibility with existing planar silicon‐based technology, make it an attractive material for novel computing devices. Recently, nonvolatile memory components made from the formation and fracture of carbon atom chains based on graphene electrodes have been reported.^[^ ^285^ ^]^ These switches are fabricated by creating nanoscale gaps using an electrical breakdown of graphene sheets. The authors proposed that conductance switching occurs through the formation of linear chains of carbon atoms that bridge the gap under a strong electric field. They further demonstrated information storage based on the concept of rank coding, in which information is stored in the relative conductance of graphene switches in a memory cell.

Moreover, the fabrication of atomic clusters can be used to achieve switch functionality, such as switches composed of binary clusters of atoms on a semiconductor surface, which have been reported to function at room temperature.^[^ ^288^ ^]^ The switching process involves the complex rearrangement of multiple atoms cooperatively. The physicochemical properties of these devices depend on their size and constituent atoms. This has led to the development of various atomic‐level devices with different functions embedded in nano‐regions, opening a new nanomanufacturing method for atomic‐level integrated electronic devices capable of operating at room temperature.

In addition to experimental work, a theoretical model for field‐assisted migration has been developed to create atomic gaps between gold nanoelectrodes using ab initio calculations.^[^ ^278^, ^289^ ^]^ This configuration evaluates gold nanostructures during tension and compression to understand the mechanism. In field‐assisted atomic migration (FAAM), the external field provides the driving force, improves the initial energy of the system, reduces the potential barrier in the migration path, and enables atomic migration. Conductance, tensile, and compressive forces serve as measurable variables in these processes, offering beneficial signals for determining the appropriate time to perform these actions.

### 7.3 Electrochemical Reaction Induced Atomic Switches

**Figure 13:**
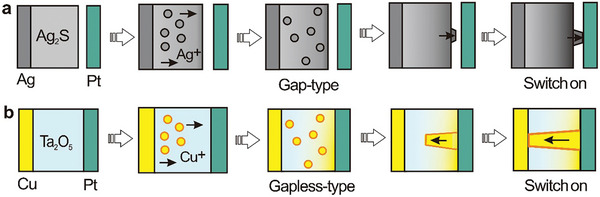
Operating mechanism of two typical atomic switches. a) Work principle of a gap‐type atomic switch. Ag, Ag_2_S, and Pt are used as a reversible electrode, an ionic‐electronic mixed conductor material, and an inert counter metal electrode, respectively. There is a gap between the mixed conductor and the counter metal electrode. b) Work principle of a gapless‐type atomic switch. Cu, Ta_2_O_5_, and Pt are used as a reversible electrode, an ionic conductive material, and a counter electrode, respectively.

For electrochemical switches, the diffusion of ions and the reduction/oxidation processes are controlled mainly by bias voltage to form/annihilate a conductive path between two electrodes. The electrochemical reaction based atomic switches can be classified as gap‐type atomic switch and gapless‐type atomic switches.^[^ ^290^, ^291^ ^]^ **Figure** **13** shows the operating mechanism of two types of atomic switches.^[^ ^292^ ^]^ When a positive bias voltage is applied to the left reversible electrode, metal cations in the ionic‐electronic mixed conductive material will migrate toward the counter electrode surface to be reduced. The reduced metal atoms precipitate at the surface to form a bridge between the ionic and electronic conductive material and the counter metal electrode, resulting in the turned‐on state with high conductance, as shown in Figure 13a. Application of a negative bias voltage causes the ionization of the precipitated metal atoms, and subsequently the ionized metal atoms are redissolved into the mixed conductor material, resulting in the annihilation of the metal atomic bridge and low conductance. Operation of the gap‐type atomic switch has been demonstrated using different materials, such as Ag_2_S, Ag–Ge containing chalcogenide, Cu_2_S, and CuI.^[^ ^271^, ^280^, ^281^, ^293^ ^]^

In contrast, for the gapless‐type atomic switch, there is no gap between the ionic conductive material and the counter electrode, as shown in Figure 13b. The counter electrode works as a blocking electrode to stop metal cations from diffusing. Metal cations (such as Cu^2+^) are reduced at the interface of ionic‐conductive‐material/counter‐electrode, which results in the precipitation of metal atoms above the counter‐electrode surface. Subsequently, the metal filament grows toward the reversible electrode. When the metal filament reaches the reversible Cu electrode, the atomic switch is turned on (high conductance). Reversing the polarity of the applied bias voltage will cause the dissolution of the metal filament, leading to the turning off of the gapless‐type atomic switch (low conductance). Different materials have been used as iron conductive materials, *e.g*., Ta_2_O_5_, hBN, and HfO_x_.^[^ ^281^, ^294^, ^295^, ^296^ ^]^

**Figure 14:**
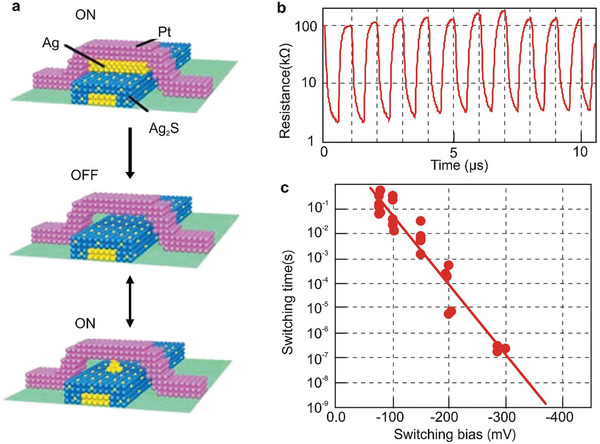
Quantized conductance atomic switch. a) Schematic diagrams of the QCAS. As‐formed switched‐on state (top), switched‐off state (middle), and switched‐on state after the initial switching‐off process (bottom). b) Experimental result of switching at 1 MHz. Alternating switching bias voltages of ±600 mV were used. c) Time taken for the resistance of the QCAS to change from an ‘off’ resistance (100 kQ) to an ‘on’ resistance was measured concerning the switching bias. Reproduced with permission from ref. [271] Copyright 2005, Springer Nature.

**Figure** **14** shows a novel gap‐type QCAS based on an electrochemical reaction.^[^ ^271^ ^]^ The tunneling current initiates a solid‐state electrochemical reaction that leads to the formation of silver nano‐protrusions on the surface of silver sulfide (Ag_2_S), which is a mixed ion and electronic conductor. The growth and shrinkage of these silver protrusions can be controlled by adjusting the polarity of the applied bias. This QCAS is composed of a pair of Ag_2_S crystal electrodes and a Pt electrode separated by ≈1 nm. When a positive bias voltage is applied to the Ag_2_S electrode, silver protrusions grow due to electrochemical reaction, forming an atomic bridge between the two electrodes. This bridge results in a significant increase in conductivity between the electrodes, effectively turning the device on. Conversely, applying a negative bias voltage to the Ag_2_S electrode causes the silver protrusion to shrink, disconnecting the atomic bridge and turning off the device, as depicted in Figure 14a.^[^ ^271^ ^]^ The QCAS can be fabricated by crossing an Ag_2_S‐coated Ag wire with a Pt wire at each intersection. To prepare the atomic‐scale gap for the atomic switch, a 1‐nm‐thick Ag layer is deposited onto the Ag_2_S‐coated silver wires, which are then crossed with platinum wires.

The initial turn‐off process requires a substantial number of Ag atoms to be ionized so that they can be incorporated into the Ag_2_S crystal. Consequently, the switching time for this first turning‐off process is relatively long, lasting several seconds. Figure 14b demonstrates the operation of the atomic switch in a crossbar structure at a 1 MHz switching frequency. Figure 14c displays the time required to switch the device from the off state, with a resistance of 100 kΩ to the on state. The results indicate that the switching time decreases exponentially as the switching bias voltage increases within the measured region. The simple structure, ease of operation, stability, and reliability of QCAS make it a promising candidate for integration into future nanodevices and novel computer architectures.

Three terminals atomic switches based on electrochemical reaction are also reported, in which the formation and annihilation of a metal filament can be controlled by a solid electrode gating,^[^ ^296^, ^297^ ^]^ light illumination,^[^ ^298^ ^]^ and electrochemical gating.^[^ ^299^ ^]^ The three‐terminal atomic switch has an advantage in that the signal and control parts are separated, which behaves like a semiconductor transistor, *i.e*., the three‐terminal atomic switch is more suitable for application to logic circuits compared to memories. Hua et al., reported an atomic field‐effect transistor constructed by integrating a metal filamentary threshold switch with a 2D MoS_2_ channel.^[^ ^296^ ^]^ In such devices, the simultaneous achievement of efficient electrostatics, very small sub‐thermionic subthreshold swings, and ultralow leakage currents make it highly desirable for next generation energy‐efficient integrated circuits and ultralow‐power applications.

**Figure 15:**
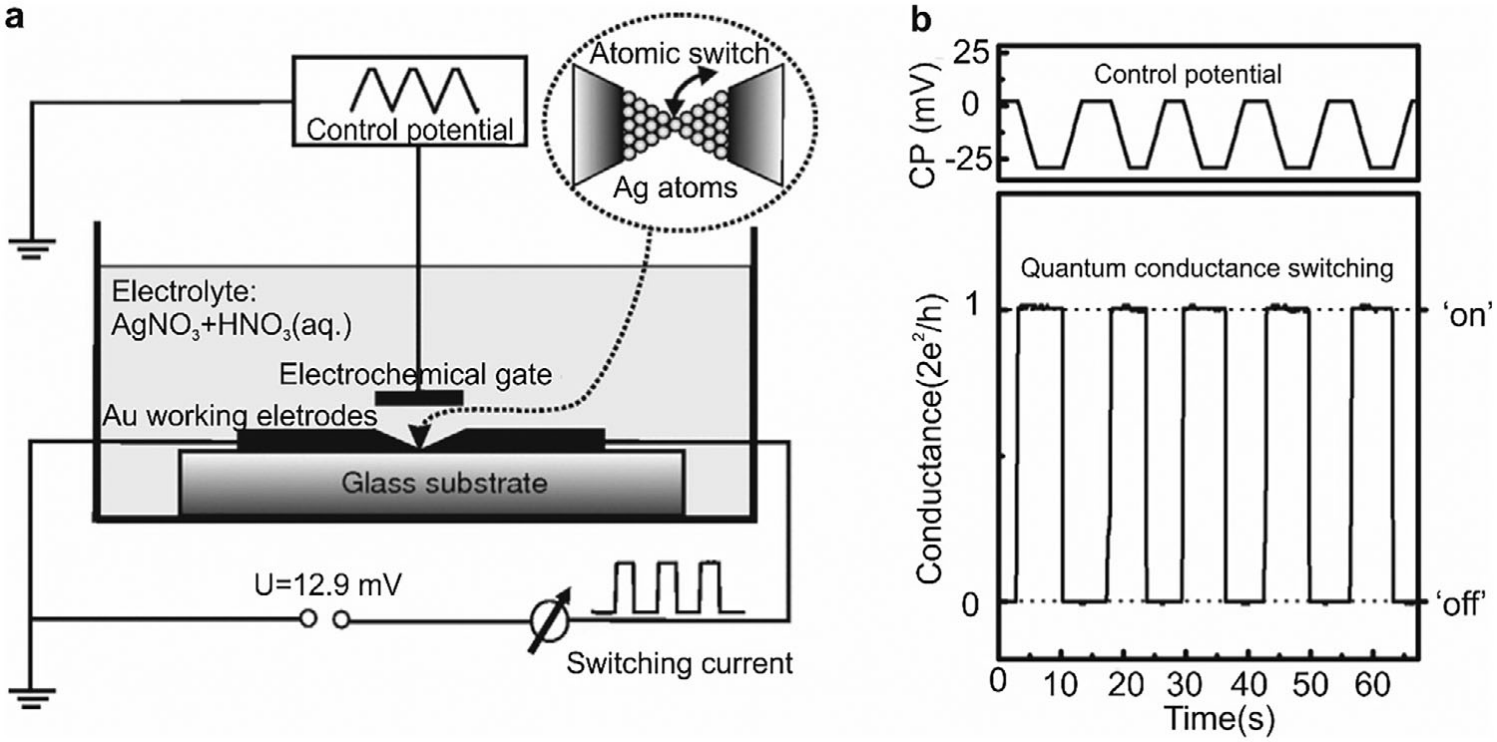
Schematic diagram of the experimental setup. a) A silver point contact is deposited electrochemically within a narrow gap between two gold electrodes on a glass substrate. b) A bistable atomic‐scale quantum conductance switch is fabricated by repeated computer‐controlled electrochemical cycling with electrochemical gating. Reproduced with permission from ref. [299] Copyright 2004, The American Physical Society.

**Figure** **15**a shows a schematic diagram of an electrochemical gated setup, which uses a gold electrode as the working electrode and a silver wire as both a counter electrode and a quasi‐reference electrode.^[^ ^299^ ^]^ The two gold electrodes are immersed in the electrolyte, and a small bias voltage is applied between the two gold electrodes with one electrode grounded. When an electrochemical potential is applied between the reference electrode and the two gold electrodes, silver islands form on the gold electrodes, which ultimately become conductive through the formation of atomic point contact. As shown in Figure 15b, the quantum conductance switch follows the electrochemical control potential, highlighting the sharp transition between the quantum conductance and tunneling states. This atomic‐level device can be operated repeatedly at room temperature, and the macro electrode or lead does not need to move to induce the switching process, offering an approach to control quantum switches and paving the way for advancements in the emerging fields of quantum electronics and atomic‐scale logic gate circuits.

Atomic switching devices are gaining attention for their potential to advance the fields of quantum electronics, atomic‐scale logic gate circuits, and energy‐saving integrated circuits. Miniaturization and continuous improvement in fabrication methods have laid the groundwork for future integration and further development.^[^ ^272^ ^]^ Despite the progress in development, atomic‐scale devices face challenges like power loss and optimization of device performance. The introduction of novel approaches, such as atomic threshold switching field‐effect transistor (ATS‐FET), which integrates a metal wire threshold switch with a 2D MoS_2_ channel, has shown promise in addressing these issues.^[^ ^296^ ^]^ The development of metal–oxide‐based atomic switches, which are fully CMOS compatible, has demonstrated a potential for high integration. These devices offer substantial potential for ultra‐low power applications, high‐efficiency static electricity, minimal sub‐threshold fluctuation, and reduced leakage current, making them a promising solution. In the future, research may focus on refining the fabrication processes to ensure the scalability and reliability of these devices. Additionally, exploring new materials, architectures, and strategies for integration with existing technologies will be crucial to fully exploit the potential of atomic switches. These devices will continue to push the boundaries of electronic miniaturization, leading to advancements in quantum electronics, atomic‐scale logic circuits, and energy‐efficient integrated systems.

## 8 Applications of Single‐Molecule/Atom Conductance Switches

Single‐molecule switches and atomic switch devices represent cutting‐edge technology in the field of nanoelectronics, with the potential to revolutionize multiple applications. These devices exploit the ability to control the position or state of individual molecules or atoms to enable precise electrical switching. Data storage, energy‐efficient electronics, molecular (or neural) computing, sensors, and nanoscale robotics are among the major applications.^[^ ^294^, ^300^ ^]^ Overall, single‐molecule switches and atomic switch devices have the potential to significantly impact a wide range of industries and applications by enabling the development of next‐generation electronics and nanoscale devices.

**Figure 16:**
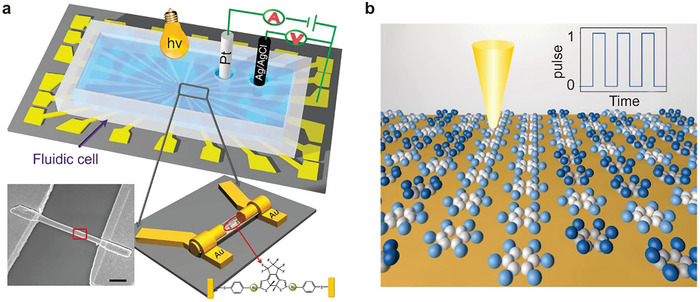
Typical applications of several molecular switch devices. a) By integrating a fluidic unit with the nanogap device, it becomes possible to create an electrochemical environment that enables the manipulation of functional molecules using optical and electrochemical stimuli. Inset: SEM image of a device fabricated by OWL‐generated nanowire (scale bar: 1 mm). Reproduced with permission from ref. [302] Copyright 2014, Springer Nature. b) Diagram illustrating how molecular memory works using a self‐assembled monolayer. A diagram of the current pulse is shown in the inset. Reproduced with permission from ref. [307] Copyright 2020, American Chemical Society.

The usage of photoisomerization molecules has enabled the development of various logic circuits such as AND, XOR (exclusive OR), INH (inhibit), half‐adder, half‐subtractor, multiplexer, demultiplexer, encoder, and decoder.^[^ ^301^ ^]^ These circuits can be formed under the irradiation of ultraviolet and visible light, allowing parallel logic operations with the same set of inputs and facilitating rapid and convenient switching (reconfiguration) among the various logic operations. Combining optical and electrochemical stimuli, diarylethene ruthenium hydrocarbon molecules can be used to implement OR gate and AND gate logic circuits. The integration of nano‐gapped devices with fluidic batteries enables the modulation of the electrochemical environment in functional molecules, as shown in **Figure** **16**a.^[^ ^302^ ^]^ This proof‐of‐concept demonstrates the potential for developing multifunctional molecular devices through rational chemical design.

For logic circuits, energy efficiency and response time are essential parameters. Using molecules as length‐controllable nano springs, Han et al., reported the design and operation of a nanoelectromechanical switch that overcomes the typical challenges of high actuation voltages and slow switching speeds.^[^ ^303^ ^]^ The molecular distance between the upper and lower electrodes can be efficiently changed by electrostatic mechanical force by applying different biases and substituting molecular systems with varying chain lengths. This allows the switching between high and low conductivity states. This method's design requires a turn‐on voltage of only 3 V and the relaxation time of the switch can reach 2 ns. This inherently modular molecular platform offers a versatile strategy for designing a variety of high‐performance, energy‐efficient electromechanical devices and low‐power logic circuits.^[^ ^303^ ^]^

Apart from their use in logic circuits, single‐molecule switching devices have several other potential applications. Researchers have developed single azobenzene molecular switch devices by using STM technology to obtain the conductance value corresponding to the *cis*‐*trans* isomer state of azobenzene molecules.^[^ ^213^ ^]^ The switching mechanism is based on the vibration excitation of local tunneling electrons, which causes the switching of the azobenzene between the *cis*‐*trans* isomers. This nano‐switching device stimulated by tunneling current offers promising prospects for future applications. Similarly, Fujii et al., demonstrated the switching effect of vinyl compounds' high and low conductance states in twisted and folded states, which may provide an essential step toward the implementation of molecular switches and memory elements in future nanotechnology.^[^ ^20^ ^]^ Additionally, by connecting molecules with azobenzene side groups to graphene electrodes, and measuring their high and low conductance states under photoelectric stimulation, researchers have developed a transistor with azobenzene side groups as electrochemical gates. These devices, combined with the photoswitching phenomenon, behave as chemically‐gated, fully‐reversible single‐molecule molecular circuits, providing new insights into constructing future practical single‐molecule devices and logic gates.^[^ ^144^ ^]^

Compared to single‐molecule devices, self‐assembled monolayer devices offer the advantages of stability and large‐area fabrication. Recent reports have demonstrated the growth of C_6_Cl_6_ molecules on Pt electrodes through both physical and chemical adsorption, enabling the physiosorbed state (“1”) and the chemisorbed state (“0”) with a moderate switching barrier.^[^ ^304^ ^]^ These well‐defined arrays exhibit thermal stability and show potential for using molecules as bits in ordered patterns for data storage, as shown in Figure 16b. First, a monolayer of C_6_Cl_6_ is created using a process called molecular beam epitaxy, which allows for precise control of the layers. Next, the memory is encoded using a pulse that modifies the adsorbed states by transferring charges between the substrates and molecules.

Similarly, atomic switch devices have been used as memory, logic devices, and programmable Switches.^[^ ^292^ ^]^ With their potential advantages of miniaturization and integration, the atomic switches could be used as nonvolatile information storage elements in the future, such as current‐driven single‐atom memory at room temperature.^[^ ^274^ ^]^ For the integration of such atomic switches into larger memory devices, a crossbar technique as demonstrated for electrochemically operated switches,^[^ ^305^, ^306^ ^]^ seems to be a promising approach. This implementation may pave the way for highly miniaturized storage devices. Furthermore, optically controlled atomic switch devices can benefit from the small focal length diameter of laser beams, enabling addressable molecular junction arrays.^[^ ^275^ ^]^ In particular, plasmons overcome the diffraction limit of light and achieve nano‐focusing. As a result, atomic switches controlled by plasmon polaritons can pave the way for highly integrated devices. Nonvolatility, low on‐resistance, and the small size of atomic switches make them suitable for use as reconfigurable switches in field‐programmable gate arrays.^[^ ^302^, ^307^ ^]^ Using the atomic switches, the size of conventional switching circuits, which currently use volatile semiconductor transistors and static random access memories, will be reduced sharply.

## 9 Summary and Outlook

In summary, the area of molecular electronics has advanced significantly with the development of several delicate systems involving molecular switches during the past few decades. This review has primarily focused on electrode materials, fabrication methods, and the detailed analysis of several typical molecular switching devices from the perspective of switching mechanisms under various stimuli. Here, we want to point out that the switch observation in one experiment may result from several possible mechanisms. A case in point is the voltage‐triggered switching. Electrostatic charging, conformational change, tilt of the molecules, and voltage‐induced breaking of the bonds at the surface may result in the conductance switch.^[^ ^308^ ^]^ Both theoretical and experimental studies may be needed to establish the origin of conductance switching.

Despite the remarkable achievements that have been made, implementing completely reversible, robust single‐molecule switches at room temperature still presents challenges. Improvements are needed in several areas, including the precise description of switching mechanisms, complex logic operations using micro‐molecules, chemical structure changes, and the uncertainty of contact between electrodes and molecules. A critical issue, the mechanism of quench effect, which blocks the reversible switching and result in a one‐way switching, is still ambiguous. Several possible mechanisms can be used to the illustrate the quenching effect: 1) Due to the specific energy level alignment between the molecule and the electrodes. When the molecule is excited from the ground state to the excited state, the energy level of its excited state may closely align with the Fermi level of gold. In such scenarios, the Fermi level of the gold electrode can influence the molecule's excited state, impeding its effective return from the excited state to the ground state and hindering reversible switching under specific conditions;^[^ ^57^ ^]^ 2) The free degree of the molecular junctions. When the molecules are embedded in a circuit, the flexibility of the molecule will be limited, which will suppress the changing of the molecule structure and thus block the molecular switching; 3) The binding of molecules to the electrode. As a molecule bonds to the electrode, the gold electrode's surface may facilitate the transfer of electrons either from the molecule to the gold surface or vice versa. This charge transfer process has the potential to impact the electron distribution of the molecule, thereby modifying its energy level structure and influencing the transition of the molecule between the ground state and exciting state; 4) The effect of bias voltage. The considerable electron will transfer between the molecule and electrode upon a bias voltage, which accompanies energy transfer. The energy transfer may result in an energy‐equilibrium system including molecule and electrode, which will suppress the energy state change of molecule; 5) The effect of the type of electrode materials. It is highly desired to clarify the underlying mechanisms with systematic experiments.

To establish high‐performance, stable, and addressable single‐molecule switching devices, it is essential to develop new novel materials, sophisticated fabrication techniques, and precise characterization methods. Sustainable growth in molecular electronics requires the introduction of new electrode materials with remarkable features and ongoing theoretical explanations. Currently, another challenge that single‐molecule devices are facing is the uncertain nature of the molecular‐to‐electrode contact interface. This interface significantly influences the device's properties. One primary goal of switching devices has always been to achieve a high switching ratio. Hence, it is imperative to investigate the lifetime of molecular excited states. The interaction between the gold electrode and the molecule may induce the mixing of the electronic state of gold with the excited state of the molecule. Such mixing could result in energy transfer, significantly reducing the excited‐state lifetime.^[^ ^309^ ^]^ Previous research has shown that incorporating metal complexes into the molecular aggregate state can raise the ground state energy by limiting the movement of the molecules in their ground state, which causes the generation of an excited state and extends its lifetime.^[^ ^310^, ^311^ ^]^ Additional approaches for molecular design^[^ ^312^ ^]^ or addressing the challenge of molecular excited‐state quenching involve efforts to modify the Fermi level of the electrode.^[^ ^313^, ^314^, ^315^ ^]^ We have enough reason to believe that single‐molecule switches and atomic switches have a bright future for bridging hard electronics to the soft molecular world.

Another critical consideration is the energy consumption required for the widespread adoption of molecular devices in the market. However, addressing overheating in microdevices is a complex and crucial challenge, particularly in the context of high‐density computing platforms utilizing microdevices such as molecular/atomic switches. Fortunately, the thermoelectric effect can be employed to manage heat in these miniaturized devices. The thermoelectric effect refers to the phenomenon of thermoelectric electromotive force and current generated due to charge diffusion caused by heat conduction when two conductors at different temperatures establish a circuit connection. This effect mainly includes three types: the Seebeck effect, the Peltier effect, and the Thomson effect.^[^ ^316^, ^317^, ^318^ ^]^ By designing molecular structures and adjusting interactions between molecules, the thermoelectric performance can be controlled to mitigate overheating issues.^[^ ^319^, ^320^, ^321^ ^]^

Thermoelectricity is poised to emerge as a pivotal energy harvesting technology for powering ubiquitous sensors and wearable devices in the future,^[^ ^322^ ^]^ offering crucial support for the sensor networks anticipated in the future Internet of Things (IoT) society.^[^ ^323^ ^]^ Numerous studies have delved into the thermoelectric effect at the microscale. For instance, Lambert et al. scrutinized the influence of different molecular structures on the Seebeck coefficient of devices,^[^ ^324^, ^325^ ^]^ thereby elucidating the mechanism for regulating the thermoelectric effect of devices through molecular design. Li et al. demonstrated a strategy for designing molecular thermoelectric junctions and devices by adjusting the morphology and surface roughness of electrodes, thereby regulating the thermoelectric effect of molecular devices.^[^ ^326^ ^]^ Reddy and coworkers performed a deeper study of both thermal and thermoelectric transport in molecular junctions, shedding light on the extremely efficient thermoelectric energy conversion in molecular junctions.^[^ ^327^, ^328^, ^329^ ^]^ The significance of thermoelectric effects in the realm of molecular electronics extends beyond energy conversion and thermal management; they also serve as valuable research tools. Through comprehensive research and the utilization of thermoelectric effects, the advancement of molecular electronics can be propelled, offering novel possibilities for the design and application of micro and nano electronic devices.

Although single‐molecule devices have made significant progress and garnered considerable interest, their practical applications face challenges as mentioned above. The utilization of large‐area junctions with self‐assembled monolayers offers potential advantages for the fabrication of practical functional devices, such as low cost, high controllability, and stability. Large area junctions are more likely to be scalable, showing excellent complementarity for single molecular devices.

## Conflict of Interest

The authors declare no conflict of interest.
